# Geographical characteristics and influencing factors of the health level of older adults in the Yangtze River Economic Belt, China, from 2010 to 2020

**DOI:** 10.1371/journal.pone.0308003

**Published:** 2024-09-13

**Authors:** Mengmeng Yang, Shengsheng Gong

**Affiliations:** College of Urban and Environmental Sciences, Central China Normal University, Wuhan, China; East China Normal University, CHINA

## Abstract

The health of older adults is crucial for the overall health of the entire life cycle. Based on population sampling survey data and census data from 131 prefecture level units in the Yangtze River Economic Belt (YREB) during 2010–2020, this study used exploratory spatial data analysis, geographical detector, stepwise regression analysis, and GTWR model to analyze the spatiotemporal pattern and influencing factors of the health level of older adults in the YREB. The results show that the health level of older adults in the YREB slightly increased from 2010 to 2020, with the most significant improvement in the upstream region and the most significant decline in the midstream region. The older adults’ health level in the YREB displays a gradient decreasing pattern of the downstream, midstream, and upstream regions. The health level of older adults in the YREB is influenced by a combination of natural and social environment factors. Areas with lower altitude and moderate humidity climates are more conducive to the health of older adults. The increase in influencing factors such as population migration rate, per capita GDP, average years of education, per capita housing construction area, per capita park green area, and green coverage rate in built-up areas is conducive to improving the health level of older adults, while lower number of health institutions per 1,000 people and higher household support rate are not conducive to improving the health level of older adults. In addition, over time, the health-promoting effect of natural environmental factors is enhanced from 2010 to 2020, and the influence of annual precipitation on shaping the spatial pattern of older adults’ health level became more obvious. Although the promoting effect of population migration on the health level of older adults tends to weaken, it remains the primary factor affecting the spatiotemporal differentiation of older adults’ health level in the YREB. The impact of social development on the health level of older adults has changed from a positive health effect (improvement) to a negative health effect (loss). The health-promoting effect of living environment factors is enhanced. The health-inhibitory effect of household support rate increased, and showed a gradient decreasing pattern from downstream to midstream to upstream. The findings of this study can provide a more in-depth understanding of the spatiotemporal pattern of the health level of older adults in the YREB and the factors influencing it, improve the health level of older adults in the region, and promote the development of healthy and active aging in the YREB, and improve the human health. At the same time, this study also supplements the related research on aging and the health level of the elderly. Firstly, it can provide reference for the research on the health of old adults in other countries and regions around the world. Secondly, it can also provide a basis for research on aging and the health of old adults in cities and counties under YREB.

## 1. Introduction

The aging of the elderly population is an undeniable global trend in the 21st century. Most regions of the planet have an aging society. Notably, the speed of China’s aging process has rapidly increased. In 2022, the proportion of senior citizens (aged 60 and above, hereinafter referred to as older adults) in China was an impressive 19.4%, an increase of 5.44% over 2010, while this proportion increased by only 3.23% between 2000 and 2010 [1]. Accelerated aging has also led to an escalation of health problems among the elderly population, as it has been reported that the number of elderly Chinese adults as who self-assessed their health status as "unable to take care of themselves" exceeded 42 million in 2020, this means that 16.6% of the total elderly population, are unable to take care of themselves [2]. In addition to the concerning state of physical health of elderly people, their mental health problems are also gradually increasing. Depressive symptoms are common among older adults in China, and a meta-analysis showed that the overall prevalence rate of depressive symptoms among older adults was 20.0% [3]. In particular, for chronic disease patients, studies have indicated that the incidence of depression among chronic disease patients in the United States and the United Kingdom is as high as 50% [4]. This is mainly because elderly chronic disease patients endure the suffering caused by the disease, and long-term treatment will invisibly increase the economic and psychological burden on older adults, which will largely reduce their sense of happiness in life and even lead to depression. Therefore, complementing and improving research on the health level of older adults can help improve human health.

Regarding the spatiotemporal patterns of the health level of older adults, demographers, sociologists and geographers have performed much research on this issue. For example, a study in China showed that the spatial differentiation of population aging in China is obvious, with the degree of aging in the southeastern region is being significantly greater than that in the northwestern region, the zonal differentiation of the east being greater than that of the west being significantly less than that of the west [5], and the urban-rural gap in the health level of older adults further widening [6]. A study of mental health problems such as suicide, anxiety, depression, and self-injury among older adults in North Carolina from 2009 to 2018 revealed that the western and northwestern regions of the state were the main high-risk clusters for suicide, anxiety, and depression, while the self-injury clusters were mainly located in the central and southeastern parts of North Carolina [7]. This article chooses the period of 2010–2020 for the research because it covers two large-scale national population censuses conducted every 10 years in China since the 21st century, which can help to better understand the spatiotemporal dynamics and influencing factors of the health level of older adults in the YREB over a long period of time.

With respect to the factors influencing the health level of older adults, previous research has shown that individual attributes (sex, age, ethnicity, disease or injury status, presence of depressive feelings, smoking habits, alcohol consumption, physical activity, participation in various activities), the natural environment (climate, topography and altitude, environmental pollution), socioeconomic factors (level of economic development, income level of residents, accessibility of medical and health resources, education, occupation, local built environment, personal living environment, marital status, social security) all influence the health of the older adults, which is broadly in line with the natural-social-biomedical model [8]. In terms of individual attributes, generally speaking, the self-rated health level of female elderly individuals is lower than that of male elderly individuals [9]. Older adults with chronic diseases, symptoms of depression, older age, lower physical activity levels, and inadequate sleep quality, as well as those who live in rural areas, have poorer self-rated health levels [10–12]. In terms of the natural environment, regions with mild climates, moderate rainfall, sufficient sunlight, good air circulation, low altitudes, and soils rich in trace elements such as molybdenum, manganese, and zinc commonly have more long-lived older adults [13]. Areas with environmental pollution are not conducive to the health of older adults [14, 15]. In addition, there are various studies on social environmental factors. A study in Switzerland showed that having a better socioeconomic status, as denoted by higher income, having supplementary insurance and a higher level of education, was associated with a better health-related quality of life among older adults [16]. A study of Turkey’s World Values Survey between 1990 and 2013 showed that low income levels and economic crises were the critical factors affecting the health and happiness of older adults [9]. An increase in medical service accessibility can weaken health inequality among the elderly population in China [17]. In recent years, studies exploring the association between exposure to green spaces and public health have increased. Urban green spaces can alleviate urban heat island effects and climate change, provide entertainment space, and promote the physical and mental health of the older adults [18]. In terms of health benefits, lower socioeconomic groups may benefit the most from the presence of green spaces [19]. "Upward" family intergenerational support has a positive impact on the physiological function of older adults, and intimate intergenerational relationships are more likely to reduce the risk of death in elderly people [20]. However, when elderly people believe themselves to be in good physical condition, they tend not to need help from others (unless they are unable to take care of themselves). In this case, family economic support increases the depression in elderly people and has a negative effect on their sense of life happiness [21]. Overall, although there have been many studies on the health of older adults, the indicators used to evaluate the health level of older adults are simplistic, and mostly one-dimensional, relying on the life expectancy of elderly people [22], the self-rated health rate of elderly people [5–16], activities of daily living (ADL), physical performance tests (PPTs) [23], the satisfaction with life scale (SWLS) [24], and Mini-Mental State Examination (MMSE) [8]. Second, the index system for measuring the factors influencing the health level of older adults is not comprehensive enough and is often limited to analysis from a particular perspective or a few facets, lacking a systematic, multifaceted index system of natural, economic, educational, medical, residential environment, family characteristics, etc. Third, the research on the health level of older adults in the YREB is mostly based on the analysis of several provinces, counties, or communities, and is mostly limited to their respective fields of demography and public health. There is no study of human geography analysis in the spatiotemporal pattern of the health level of older adults and its influencing factors in the YREB.

**Fig 1:**
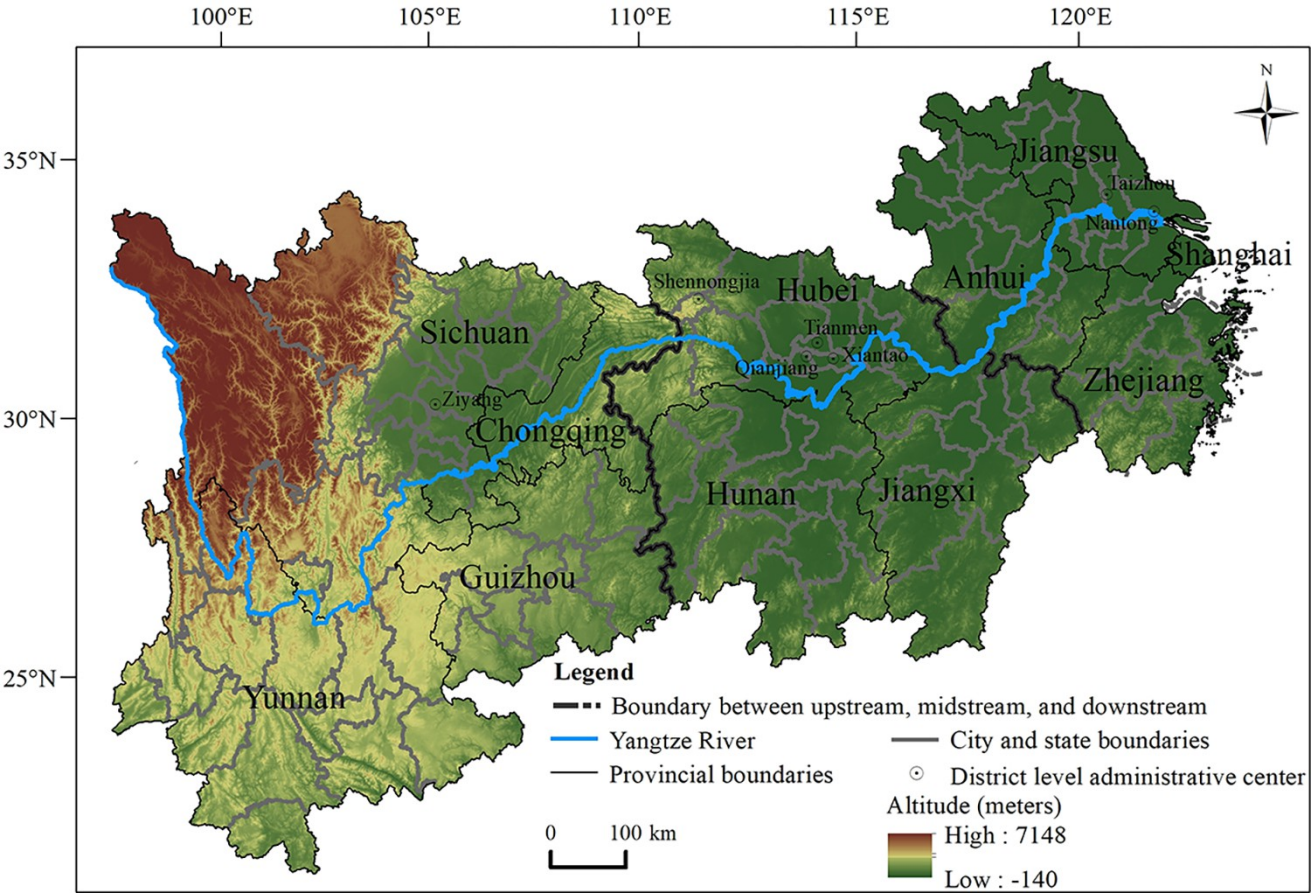
Overview of the Yangtze River Economic Belt. Note: The basemap was obtained from the United States Geological Survey (https://apps.nationalmap.gov/services/), and the map boundary has not been changed. Cartographic software: ArcGIS.

The Yangtze River Economic Belt (YREB) is an influential strategy in China’s overall national development [25]. It spans the three key regions of East, Central, and West China, encompassing 11 provinces and cities, including Shanghai, Jiangsu, Zhejiang, Anhui, Jiangxi, Hubei, Hunan, Chongqing, Sichuan, Yunnan, Guizhou, etc., and covers an area of 2.0523 million square kilometers, approximately 21.4% of China’s land area (Fig 1), and the population and GDP exceed 40% of the country. The results of the seventh National Population Census showed that the aging populations of Nantong, Taizhou, and Ziyang reached high values of 22.67%, 22.01%, and 22.62%, respectively. The aging population of the YREB is constantly increasing, and there are significant differences in the degree of the aging population among provinces and cities. Previous papers [26–30] about the health of older adults in the YREB have focused mostly on individual provinces or counties, with more attention given to the field of epidemiology or sociology. There has yet to be a study on the spatiotemporal pattern and influencing factors of the health level of older adults in the YREB. Hence, it is necessary and meaningful to study the health level of the elderly population in the YREB. From the perspective of health geography, this article, based on population sampling survey data and census data from 2010 to 2020, takes 131 prefecture-level units in the YREB as the research object, uses exploratory spatial data analysis, geodetector, stepwise regression analysis, and spatiotemporal geographically weighted regression to analyze the spatiotemporal characteristics and influencing factors of the health level of older adults in the YREB. The findings of this study can provide a more in-depth understanding of the spatiotemporal pattern of the health level of older adults in the YREB and the factors influencing it, improve the health level of older adults in the region, and promote the development of healthy and active aging in the YREB. At the same time, it is hoped that this study will provide a reference for other countries or regions to solve the problem of aging to promote the sustainable development of the population, economy and society of these countries or regions.

## 2. Data and methods

### 2.1 Data

#### 2.1.1 Variables

*(1) The index selection of the health level of older adults*. According to international guidelines, people over the age of 60 are classified as elderly people. There have been numerous studies that have evaluated the health status of older adults from physiological, psychological, and social perspectives, among others. In measuring the health status of older adults, studies often used single indicators such as the life expectancy of the elderly, the self-rated health rate of the elderly, activities of daily living (ADL), physical performance tests (PPTs), the satisfaction with life scale (SWLS), and Mini-Mental State Examination (MMSE), etc. However, it should be noted that these indicators only highlight one dimension of the health level of older adults, whether subjective or objective, physiological or psychological. The Almaty Declaration of World Health Organization (WHO) defines health as "the absence of disease and weakness, and the fullness of physical, mental, and social well-being". Therefore, inspired by the World Health Organization’s "Global 100 Core Health Indicators" and the key indicators of the "Healthy China 2030" planning outline, combined with substantial sociological, demographic research [5–31], and our research objectives, based on the principles of scientificity, objectivity, and operability, a comprehensive evaluation index system for the health level of older adults is constructed using three indicators: life expectancy of the elderly, self-rated health rate of the elderly, and disability rate of the elderly (Table 1). Among them, life expectancy of the elderly refers to the average life expectancy of the 60–64 age group, which is a traditional indicator for measuring the health of older adults and can be used to reflect their objective health status. The life expectancy of the elderly is based on the population and mortality data by age and sex in each level of unit, calculated using a simplified life table [32]. The self-assessment data on the health status of elderly people is a survey data conducted by the Chinese government in the form of self-assessment of elderly people in the national population census. The self-assessment health status survey of elderly people includes four options: healthy, basic health, unhealthy but able to take care of themselves, unhealthy and unable to take care of themselves. Elderly people make choices based on their health status among the four options. In this study, the self-rated health rate of the elderly and disability rate of the elderly were calculated based on these data of the self-assessment health status survey of elderly people, which are mainly used to reflect the psychological health status of the elderly population [5]. Wherein, the self-rated health rate of the elderly refers to the proportion of the elderly population aged 60 and above who are "healthy" and "basic health". The disability rate of the elderly is obtained by calculating the proportion of elderly people who self-assess their health status as "unhealthy and unable to take care of themselves" to the total number of elderly people aged 60 and over. "Unhealthy and unable to take care of oneself" refers to poor health status within the last month and the inability to perform activities of six basic activities of daily living (ADLs), such as toileting, dressing, eating, walking, bathing and transferring indoors, which is basically the same as the definition of disabled elderly, so it is defined as the disability rate of the elderly, as shown in Table 1. Overall, life expectancy of the elderly objectively encapsulates the health status of this population group. Data on the self-assessed health status of older adults, including the self-rated health rate of the elderly and disability rate of elderly people, are critical indicators for assessing the psychological health level of elderly people. A harmonization of objective assessments coupled with subjective perceptions, as well as a consideration of physiological and psychological factors, can provide a more holistic and comprehensive reflection of the health status of older adults in a given country or region.

**Table 1:** Indicators and data sources for the health level and influencing factors of older adults.

Variable factors	Index	The significance of indicators	Data sources
The health level of older adults	Life expectancy of the elderly (Year)	Calculated through a simplified life table.	*2010 Population Census Yearbook*, and *2020 Population Census Yearbook* from various provinces and cities.
	Self-rated health rate of the elderly (%)	Percentage of seniors aged 60 years and older who rated their health as "healthy" or "basic health" as a percentage of the total elderly people.	
	Disability rate of the elderly (%)	The disability rate of the elderly is calculated by determining the proportion of elderly persons with a self-rated health status of "unhealthy and unable to take care of themselves" among persons aged 60 years and over in the total elderly people.	
Natural environment	Annual average temperature (*x*_*1*_/°C)	The arithmetic mean value of the monthly average temperature for each month of the year.	China Meteorological Data Network’s Annual Ground Climate Standard Values Dataset for China.
	Annual precipitation (*x*_*2*_/mm)	The total amount of monthly precipitation throughout the year.	
	Average altitude (*x*_*3*_/m)	The average altitude of prefecture-level cities.	Computer Network Information Center of the Chinese Academy of Sciences’ China 250m resolution digital elevation data.
Population migration	Population migration rate (*x*_*4*_/%)	Net migration population (difference between permanent population and registered resident population) as a percentage of permanent population.	*2010 Population Census Yearbook* and *2020 Population Census Yearbook* of each province and city.
Social development level	Per capita GDP (*x*_*5*_/Yuan/person)	Take logarithmic values. Used to reflect the economic level of the region.	*Statistical Yearbook*, *Health Statistical Yearbook*, *2010 Population Census Yearbook*, *2020 Population Census Yearbook*, National Economic and Social Development Statistical Bulletin of each province and city.
	Number of health institutions per 1,000 people (*x*_*6*_/Number)	The number of different medical and health institutions per thousand permanent residents. Used to reflect the medical and health status of the region.	
	Average years of education (*x*_*7*_/Year)	The average of the total years of education received by the population aged 6 and over. Used to reflect the educational level of the region.	
Living environment	Per capita housing construction area (*x*_*8*_/ Square meters per person)	The total building area of housing owned by each person on average.	*Statistical Yearbook of Urban Construction in China*, *Statistical Yearbook* of Various provinces and Cities, Statistical Bulletin on National Economic and Social Development.
	Per capita park green area (*x*_*9*_/ Square meters per person)	Average public green area occupied by each person.	
	Green coverage rate in built-up areas (*x*_*10*_/%)	Percentage of green coverage area in urban built-up areas.	
Family characteristics	Household support rate (*x*_*11*_/%)	Percentage of elderly population supported by other family members in total elderly population.	*2010 Population Census Yearbook* and *2020 Population Census Yearbook* of each province and city.

*(2) The index selection of influencing factors*. The geographic environment is closely related to human health. Theoretically, the health of the human body mainly depends on three major elements: biological heredity, natural environment, and social environment. Biological heredity belongs to the fields of medicine and biology. In this article, we explore the effects of natural environment and social environment factors on the health level of older adults from the perspective of health geography.

Natural environment factors are fundamental contributors to human health and have a consistent impact on individual well-being. Research showed that the mortality rate of older adults has a temperature threshold and increases when temperatures fall below or exceed that threshold [33]. At the same time, extreme weather conditions have been found to increase mortality rates among older adults and affect their health, such as when extreme cold increases the risk of ADL disability and mortality among older adults, and extreme heat can lead to cognitive impairment in seniors [34, 35]. The temperate and humid climate is conducive in reducing the incidence of chronic diseases among the elderly population, while severe precipitation weather conditions (floods), not only directly endanger public health, but also increase the risk of vector-borne, viral respiratory, gastrointestinal, and other diseases, rendering the elderly demographic susceptible to infection [36]. Chronic exposure to high-altitude environments may impact cardiovascular health, disease development and life-expectancy [37]. The mortality rates of coronary heart disease and stroke are relatively low in high-altitude areas of Switzerland [38]. But rapidly rising to high altitudes may adversely effects on the cardiovascular health of lowland people, especially those with previous illnesses. Therefore, this study selected annual average temperature (*x*_*1*_), annual precipitation (*x*_*2*_) and average altitude (*x*_*3*_) as the basic variables that serve as indicators of natural environmental factors that influence the health level of older adults.

Social environment factors include economic conditions, medical standards, education levels, population migration, and personal living environments, etc. Regional disparities in development may lead to spatial disparities in the health status of the elderly [6–39]. A study on the health status of elderly Chinese adults found that the health status of older adults in eastern China exceeded that in central and western regions, and that in urban areas outpaces that in rural areas [6]. Widening income disparities may reduce the subjective well-being of older adults, especially those in rural areas, which may lead to inequality in their mental health [9]. In all, it has been observed that the health status of older adults tends to be more favorable in regions with advanced levels of economic development, abundant health resources and improved access to health care, as well as extensive access to higher education, tend to be more favorable [31]. The large-scale population migration resulting from inter-regional differences in social development can directly exacerbate the spatial inequality in population health. First, population migration induces transformative changes in population structure in both the inflowing and outflowing areas. Second, population migration indirectly modulates the socioeconomic development, utilization rate of public medical resources, conventional family care models, and other factors in both the inflowing and outflowing areas, thus increasing the spatial disparity of population health [40]. In addition, improving the comfort of the residential environment could improve population health. Research has indicated that housing is a significant factor influencing the emotional and physical health of the population, high housing costs and frequent relocation may increase the financial burden on the population, thereby affecting their health status [41]. Poor-quality housing (such as limited living space, incomplete or outdated housing facilities, lack of indoor toilets, poor lighting, etc.) exert an indirect negative influence on the mental and physical health of older adults [27, 42]. Green spaces have a significant and lasting impact on the mental health level of residents [22]. Given the physical limitations of older adults, parks serve as a primary outdoor place for them. As an integral part of our urban green spaces, park landscapes exhibit versatility, are equipped with essential facilities, and offer ample independent and open spaces where seniors can engage in diverse recreational activities [43]. This contributes considerably to improving the health of the elderly people. Dissatisfaction with green spaces will also have a negative impact on the self-rated health levels of older adults due to its effect on their well-being [44]. Studies showed that the economic status of older adults has an undeniable impact on their health, and having sufficient economic resources is beneficial for their physical and mental health, so the self-rated health conditions of financially independent seniors generally show better outcomes than those dependent on family members for sustenance [45]. In summary, this article selected population migration rate (*x*_*4*_), per capita GDP (*x*_*5*_), number of health institutions per 1,000 people (*x*_*6*_), average years of education (*x*_*7*_), per capita housing construction area (*x*_*8*_), per capita park green area (*x*_*9*_), green coverage rate in built-up areas (*x*_*10*_), and household support rate (*x*_*11*_) as social environment factors affecting the health level of older adults (Table 1).

#### 2.1.2 Data sources

*(1) The health level of older adults*. The population indicators of the local units come mainly from statistical yearbooks, population census data (Table 1).
*(2) Influencing factors*. The data used in this study are from statistical yearbook, health statistical yearbook, statistical yearbook of urban construction in China, 2010 population census yearbook, 2020 population census yearbook, national economic and social development statistical bulletin, and China meteorological data network’s annual ground climate standard values dataset for China (https://data.cma.cn/) of each province and city. The 250m resolution digital elevation data of the YREB is derived from the geospatial data cloud of the Computer Network Information Center of the Chinese Academy of Sciences (https://www.gscloud.cn/).

#### 2.1.3 Administrative divisions

In this study, Shanghai and Chongqing, which are under the jurisdiction of the YREB, as well as Xiantao city, Qianjiang city, Tianmen city and Shennongjia Forest District in Hubei Province, are considered as prefecture-level units (Fig 1). Therefore, the YREB has a total of 131 prefecture-level units. Among them, the upstream region includes 47 prefecture-level units under the jurisdiction of the four provinces and municipalities of Yunnan, Guizhou, Sichuan and Chongqing. The middle-stream region includes 42 prefecture-level units under the jurisdiction of the three provinces of Hubei, Hunan and Jiangxi. The downstream region includes 42 prefecture-level units under the jurisdiction of the four provinces and municipalities of Jiangsu, Zhejiang, Anhui and Shanghai.

### 2.2 Methods

#### 2.2.1 Comprehensive method for measuring the older adults’ health level

This article comprehensively measures the health level of older adults using three indicators: life expectancy of the elderly, self-rated health rate of the elderly, and disability rate of the elderly. First, the data are standardized to eliminate the influence of data dimensionality and variation range; second, the entropy method [46] is used to determine the weights of the three health indicators; finally, the weighted sum method is used to calculate the health level of older adults.

*① Data standardization processing*. The life expectancy of the elderly and the self-rated health rate of the elderly are positive indicators, while the disability rate of the elderly is a negative indicator. The minimum and maximum range normalization methods are used for dimensionless processing, respectively.
*② Entropy weighting method*. The entropy method is a method of objectively weighting indicators based on the size of their information entropy. The smaller the information entropy, the greater the degree of dispersion of the indicator. The more information it contains and transmits, the greater the weight assigned.

Calculate the proportion of the *j*-th indicator value in the *i*-th prefecture-level unit:

$$Y_{ij}=\frac{X_{ij}}{\sum_{i=1}^{m}X_{ij}}$$ (1)

Calculate the entropy value of each indicator:

$$E_{j}=\frac{\sum_{i=1}^{m}\left(Y_{ij}\;lnY_{ij}\right)}{lnm}$$ (2)

Calculate the weights of each indicator:

$$W_{j}=\frac{1-E_{j}}{\sum_{j=1}^{n}\left(1-E_{j}\right)}$$ (3)

③ Using weighted sum method to calculate the health level of older adults:

$$H_{ij}=\sum_{j=1}^{3}X_{ij}W_{j}$$ (4)

In the formula, *x*_*ij*_ represents the original value of index *j* for unit *i* at the prefecture level; *x*_*j max*_、*x*_*j min*_ represent the maximum and minimum values of all prefecture-level units under index *j*, respectively; *X*_*ij*_ represents the standardized value of index *j* for unit *i* at the prefecture level; *m* represents the total number of inland units in the study area (*m* = 131 in this article); *n* represents the number of indicators (*n* = 3 in this article); *E*_*j*_ represents the entropy value of index *j*; *W*_*j*_ represents the weight of indicator *j*; *H*_*ij*_ represents the health level of older adults in indicator *j* of unit *i* at the prefecture level. After calculation, the weights of life expectancy of the elderly, self-rated health rate of the elderly, and disability rate of the elderly determined by the entropy method are 0.334, 0.333, and 0.333, respectively.

#### 2.2.2 Analysis method of spatiotemporal pattern of older adults’ health level

*(1) Exploratory Spatial Data Analysis (ESDA)*. This method can be used to describe and visualize the spatial distribution characteristics of data and visualize them, identify outliers in spatial data, reveal the spatial structure and spatial interaction mechanism of geographical phenomena, and is a collection of a series of spatial data analysis methods and technologies. Based on Arc-GIS software, ESDA mainly includes global spatial autocorrelation analysis, local spatial autocorrelation analysis, and Getis-Ord Gi* index. This article mainly used global spatial autocorrelation analysis and Getis-Ord Gi* index to reveal the spatial distribution pattern of the health level of older adults in 131 prefecture-level units of the YREB from 2010 to 2020.

Global spatial autocorrelation is used to measure the overall spatial correlation degree of the health level of older adults in the YREB, Moran’s *I* is commonly used to measure, with a value range of [–1,1]. If Moran’s *I* is greater than 0 and less than 1, it indicates the existence of positive spatial correlation, which is an agglomerative distribution. If Moran’s *I* is greater than -1 but less than 0, it indicates the presence of a negative spatial correlation, which is a discrete distribution. If Moran’s *I* is equal to 0, then there is no spatial correlation [47]. It is calculated as follows:

$$I=\frac{n\sum_{i=1}^{n}\sum_{j=1}^{n}w_{ij}(x_{i}-\underline{x})(x_{j}-\underline{x})}{\sum_{i=1}^{n}\sum_{j=1}^{n}w_{ij}\sum_{i=1}^{n}{\left(x_{i}-\underline{x}\right)}^{2}}$$ (5)

In (5), *n* = 131, indicates 131 prefecture-level units of the YREB; *w*_*ij*_ represents the weight matrix; *x*_*i*_ and *x*_*j*_ indicate the health level of older adults in county *i* and county *j*, respectively; and *x* represents an average value. The significance of spatial autocorrelation is assessed by the standardized statistic *Z*. The formula is as follows:

$$Z=\frac{I-E(I)}{\sqrt{VAR(I)}}$$ (6)

Generally, according to the normal distribution test value, when *Z* is greater than 1.96 or *Z* is less than -1.96(α = 0.05), there is a significant spatial correlation with respect to the health level of older adults in the YREB.

The Getis-Ord Gi* (abbreviated as Gi* below) index is used to measure the local spatial distribution characteristics of the older adults’ health level in the YREB, which can be used to identify the spatial distribution of hot and cold point regions. The formula is as follows:

$$G_{i}^{*}=\sum_{j=1}^{n}W_{ij}x_{j}/\sum_{j=1}^{n}x_{j}$$ (7)

In (7), *n*, *x*_*j*_, and *w*_*ij*_ have the same meaning as in Eq 5. When Gi* > 0 indicates the presence of hot spots, Gi* < 0 indicates the presence of cold spots.

*(2) Center of gravity model*. The regional center of gravity is the point at which the average value of a geographic element reaches equilibrium in spatial moments. Based on Arc-GIS software, the center of gravity model is used to analyze the orientation and migration trajectory of the center of gravity of the health level of older adults in the Yangtze River Economic Zone from 2010 to 2020.

#### 2.2.3 Analysis method of influencing factors of older adults’ health level

*(1) Geographical detector (GD)*. A geographical detector (GD) includes four detectors: single-factor detection, interaction detection, risk area detection, and ecological detection. Among them, single-factor detection measures the explanatory power of the indicator factor X on the comprehensive index Y by comparing the cumulative variance of each subregion with the variance of the entire study area, which can effectively detect the spatial differentiation of geographical phenomena and their drivers. In this paper, single-factor detection is used to identify the influencing factors on the health level of older adults in the YREB. The calculation formula is as follows:

$$q=1-\frac{\sum_{h=1}^{L}N_{h}\sigma_{h}^{2}}{N\sigma^{2}}$$ (8)

In (8), *q* is the decisive indicator for measuring the factors influencing the health level of older adults. The value range of *q* is [0,1], and the larger the value of *q*, the stronger the explanatory power of this factor on the spatial distribution of older adults’ health level in the YREB. Conversely, the weaker the explanatory power, and *q* = 0 indicates that this factor is not related to the spatial distribution of older adults’ health level in the YREB; *h* = 1,… *L* is the number of subregions in the classification of influencing factors; *N*_*h*_ is the sub-district *h*, *N* is the number of spatial units, where *N* = 131, which is the 131 cities in the YREB; $\sigma_{h}^{2}$ and *σ*^2^ represent the discrete variance of the health level of older adults in subregion *h* and the YREB, respectively.

*(2) Stepwise regression analysis*. Stepwise regression analysis is commonly used to solve the problem of collinearity among independent variables. In this paper, stepwise regression analysis is utilized to examine the strength of the factors influencing the health level of older adults in the YREB. The procedure is as follows: initially conduct a simple regression between the independent variable and the dependent variable, select the optimal model based on the significance of the independent variable and the goodness of fit of the equation, and use it as the base model. Subsequently, gradually add more independent variables to the base model and perform an *F*-test on the model to test the significance and collinearity of all independent variables. Variables that fail the test are eliminated until all potential variables are included. After stepwise regression, the independent variables retained in the model are both the most important and non-collinearity.
*(3) Spatio-temporal geographically weighted regression (GTWR)*. As an enhancement to the traditional geographically weighted regression (GWR) for cross-sectional data, GTWR introduces the time dimension to comprehensively utilize the geographical information from both time and space, effectively improving the regression results, more accurately reflecting the relationship between spatial data, effectively avoiding the influence of spatial autocorrelation, and thus improving the accuracy and reliability of the model [6]. In this article, GTWR is used to analyze the spatial differentiation of factors affecting the health level of older adults in the YREB in different years. The formula is as follows:

$$Y_{i}=\beta_{o}(u_{i},v_{i},t_{i})+\sum_{k=1}^{P}\beta_{k}(u_{i},v_{i},t_{i})X_{ik}+\varepsilon_{i};\;i=1,2,\ldots ,n$$ (9)

In (9), *Y*_*i*_ is the health level of the elderly population in the *i*-th city, *n* is the number of cities (n = 131 in this article), *u*_*i*_, *v*_*i*_, *t*_*i*_ represent the spatiotemporal coordinates of the *i*-th city, *β*_*o*_(*u*_*i*_, *v*_*i*_, *t*_*i*_) represents the constant term of the regression, and *P* is the total number of variables; *X*_*ik*_ represents the *k*-th value of the independent variable of the *i*-th city; *β*_*k*_(*u*_*i*_, *v*_*i*_, *t*_*i*_) represents the regression coefficient of the *k*-th independent variable of the *i*-th city; *ε*_*i*_ is the error term.

## 3. Spatiotemporal pattern of older adults’ health level

### 3.1 Temporal changes of older adults’ health level

Over the past 10 years, the health level of older adults in the YREB slightly increased, manifested as 0.588 in 2010, and 0.592 in 2020, with an amplitude of 0.004. Specifically, the health level of older adults in the upstream region gradually improved from 0.507 in 2010 to 0.556 in 2020, with an amplitude of 0.049. The health level of older adults in the midstream region declined from 0.593 in 2010 to 0.564 in 2020, with an amplitude of 0.029. The health level of older adults in downstream areas slightly decreased from 0.675 in 2010 to 0.661 in 2020, with an amplitude of 0.014 (Table 2). It can be seen that in the past decade, the health level of older adults in the YREB has slightly improved, with the most significant improvement in the upstream region and the most significant decline in the midstream region.

**Table 2:** Temporal changes of older adults’ health level in the Yangtze River Economic Belt.

Time	The Yangtze River Economic Belt	The upstream region	The midstream region	The downstream region
2010	0.588	0.507	0.593	0.675
2020	0.592	0.556	0.564	0.661

### 3.2 Spatial distribution of older adults’ health level

**Fig 2:**
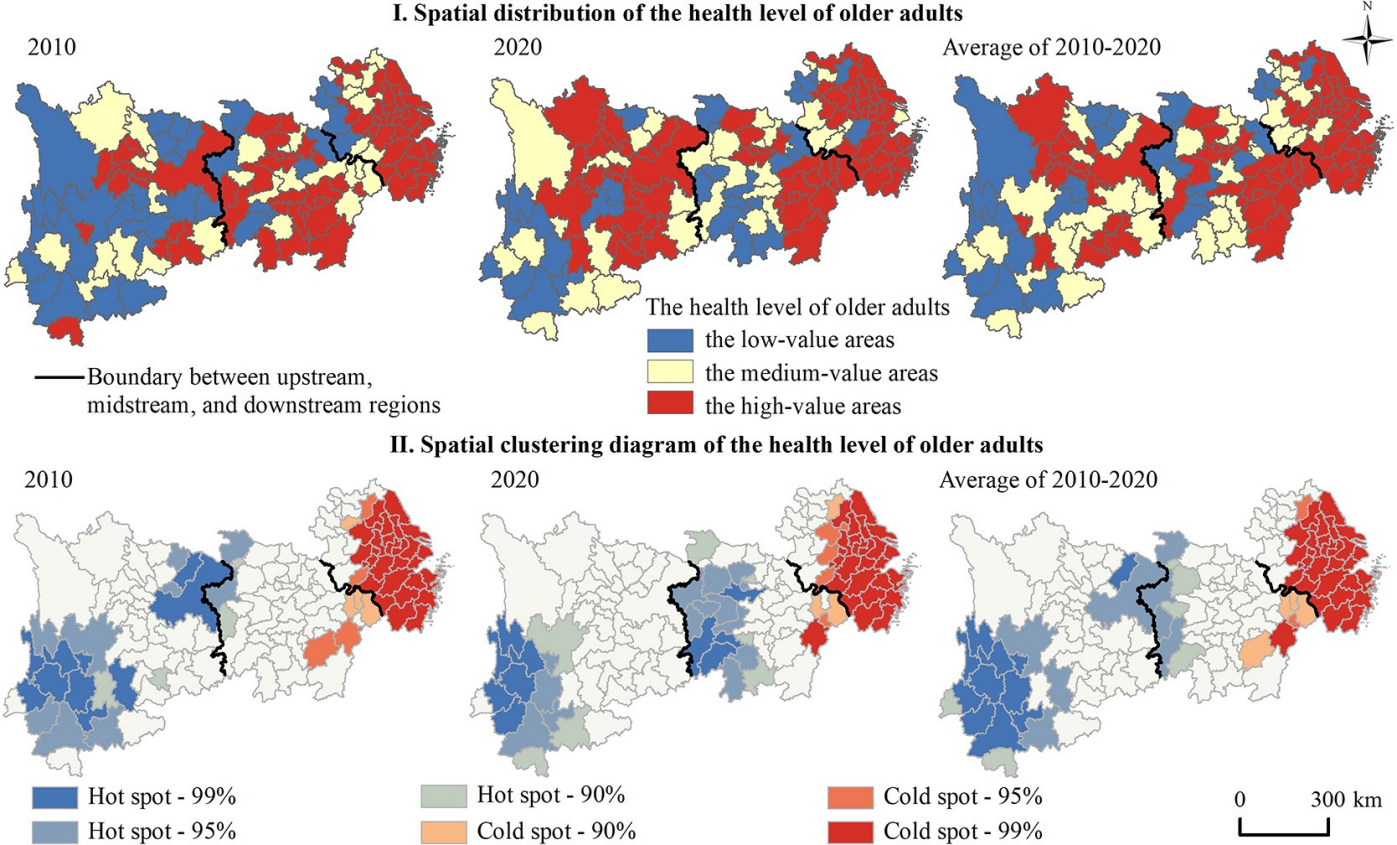
Spatiotemporal pattern evolution of the health level of older adults in the Yangtze River Economic Belt. Note: The basemap was obtained from the United States Geological Survey (https://apps.nationalmap.gov/services/), and the map boundary has not been changed. Cartographic software: ArcGIS.

From 2010 to 2020, the health level of older adults in the YREB was categorized into three levels according to the natural breakpoint method, and named as the low-value areas (0–0.4926), the medium-value areas (0.5926–0.5910), the high-value areas (0.5910–0.9626). As shown in Fig 2, in 2010, the number of high-value areas was 66, accounting for 50.38% of the total number of prefecture-level units. Among them, there were 13, 24, and 29 high-value areas in the upstream, midstream, and downstream regions, accounting for 9.92%, 18.32%, and 22.14% of the total number of prefecture-level units, respectively, forming a gradient decreasing pattern in the downstream, midstream, and upstream areas. In 2020, the number of high-value areas in the YREB increased to 69, accounting for 52.67% of the total number of prefecture-level units. Among them, the proportion of high-value areas in the upstream, midstream, and downstream areas were 16.79%, 12.98%, and 22.90%, respectively. Notably, the health level of older adults in the downstream region remained the highest, the health level of older adults in upstream region significantly improved, while the midstream experienced considerable declined. This phenomenon was attributed to the significant decline in Hunan and Hubei provinces. Overall, from 2010 to 2020, the number of high-value areas in the YREB is 67, accounting for 51.15% of the total number of prefecture-level units. Specifically, there are 17 high-value districts in the upstream, 20 in the midstream, and 30 in the downstream regions, accounting for 12.98%, 15.27%, and 22.90% of the total number of prefecture-level units, respectively. It can be seen that there is spatial difference in the health level of older adults among the upstream, midstream and downstream regions, with the health level of older adults in the downstream region being higher than that in the midstream and upstream regions as a whole.

### 3.3 Spatiotemporal evolution of older adults’ health level

#### The hot spots of older adults’ health level in the YREB from 2010 to 2020 were gathered in midstream and downstream regions, while the cold spots were mainly distributed in Yunnan, Hubei, and Hunan provinces, and showed a tendency to migrate from upstream to midstream region

Global spatial autocorrelation analysis showed that the Moran’s *I* of the health level of older adults in the YREB varied from 0.42 in 2010 to 0.37 in 2020, all of which passed the significance test, indicating a clustered distribution. Getis-Ord Gi* index can be performed, and the results are shown (Fig 2): In 2010, the hot spots were mainly concentrated in Jiangsu, Zhejiang, Anhui, and Jiangxi provinces of the midstream and downstream regions, with a total of 36 cities. Cold spots were mainly distributed in Yunnan, Chongqing, and Hubei provinces of the upstream and midstream regions, with a total of 23 cities. In 2020, the footprint of hot spots shrunk and was primarily concentrated in 34 cities in the midstream and downstream regions. The scope of cold spots in Yunnan Province decreased (12 cities), and new cold spots (19 cities) appeared in Hubei and Hunan provinces of the midstream region. Throughout the 2010–2020 study period, the hot pots of older adults’ health level in the YREB were predominantly located in Zhejiang, Shanghai, Jiangsu, Anhui, and Jiangxi provinces of midstream and downstream regions, while the cold spots were mainly distributed in Yunnan, Hubei, and Hunan provinces, exhibiting a trend of migration from upstream to midstream. This indicates that the health level of older adults in the downstream region has always been relatively higher, and the health level of older adults in upstream region has significantly improved. Contrarily, the health level of older adults in the midstream region of Hunan and Hubei provinces considerably declined, showing a "low-lying" characteristic in midstream region.

**Fig 3:**
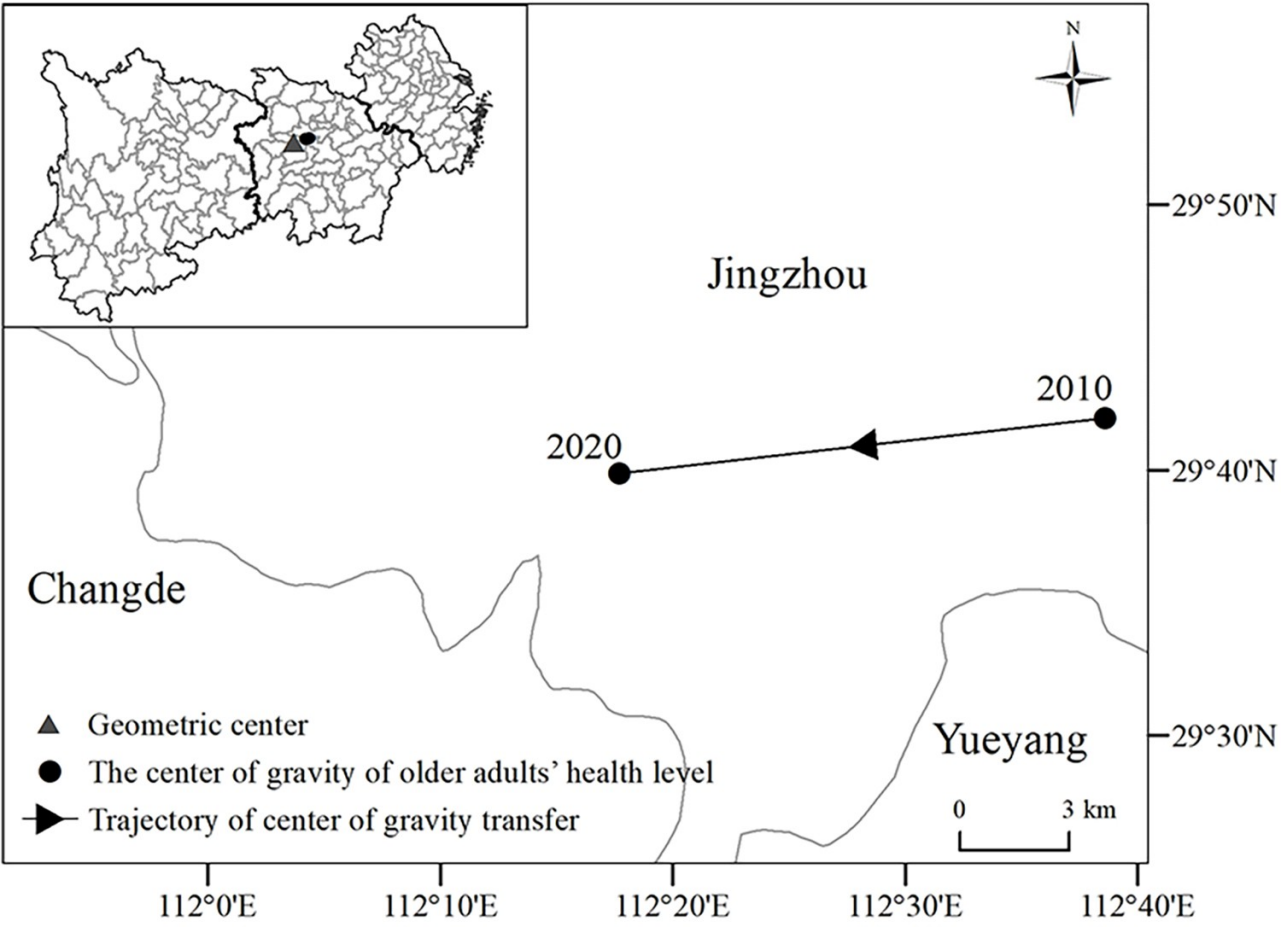
Migration map of the center of gravity of older adults’ health level in the Yangtze River Economic Belt. Note: The basemap was obtained from the United States Geological Survey (https://apps.nationalmap.gov/services/), and the map boundary has not been changed. Cartographic software: ArcGIS.

From 2010 to 2020, the center of gravity of the health level of older adults in the YREB has been swinging back and forth to the east of the geometric center, and gradually shifted to the southwest (Fig 3). It can be determined that the health level of older adults in the downstream region was relatively higher than that of the upstream and midstream regions during the study period, and in recent years, there has been a significant improvement in the health level of older adults in the upstream region.

## 4. Influencing factors of older adults’ health level

### 4.1 Effect intensity of influencing factors

Based on the data of 11 influencing factors in five dimensions, namely, the natural environment, population migration, social development level, living environment, and family characteristics, geographical detector and stepwise regression analyses were used to explore the intensity of factors influencing the health level of older adults in the YREB.

**Fig 4:**
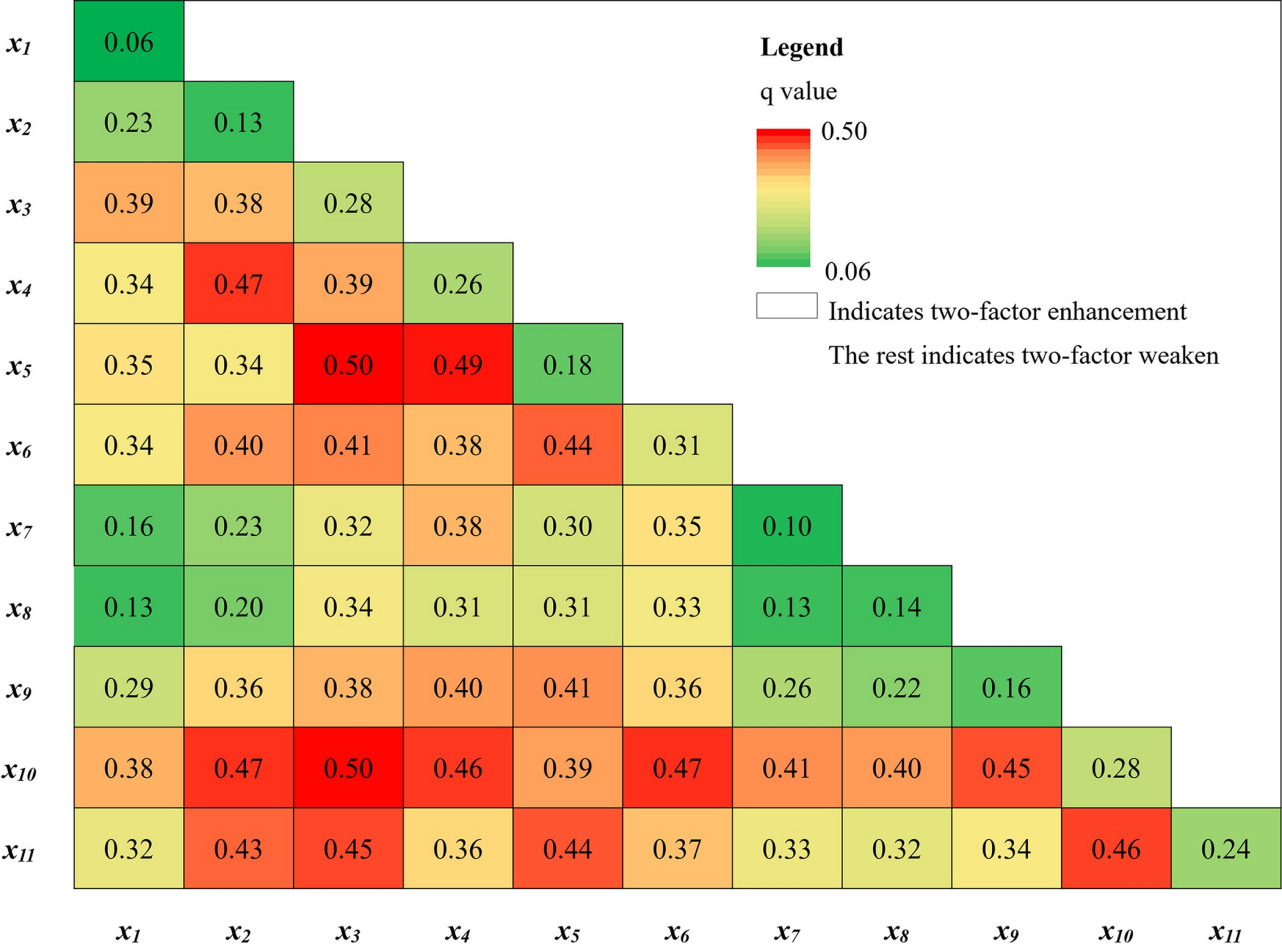
Interaction hotspot maps of influencing factors on older adults’ health level in the Yangtze River Economic Belt. Note: *x*_*1*_: average annual temperature, *x*_*2*_: annual precipitation, *x*_*3*_: average altitude, *x*_*4*_: population migration rate, *x*_*5*_: per capita GDP, *x*_*6*_: number of health institutions per 1,000 people, *x*_*7*_: average years of education, *x*_*8*_: per capita housing construction area, *x*_*9*_: per capita park green area, *x*_*10*_: green coverage rate in built-up areas, *x*_*11*_: household support rate.

(1) Geographical detector was used to detect the factors influencing the health level of older adults in the YREB, and the results showed that, in terms of single-factor detection, only the average annual temperature did not pass the *p* < 0.1 significance test. The following factors were ranked by *q*-value: average years of education (0.31) > population migration rate (0.28) > average altitude (0.28) > per capita GDP (0.26) > household support rate (0.24) > number of health institutions per 1000 population (0.18) > green coverage rate in built-up areas (0.16) > per capita park green area (0.14) > annual precipitation (0.13) > per capita housing construction area (0.10). revealing that the single factor explanatory power of socio-economic factors is stronger. In terms of interaction detection, the interaction between socio-economic factors and natural meteorological factors is significantly stronger, such as interaction between average altitude and number of health institutions per 1000 population (0.50), average altitude and population migration rate (0.50), annual precipitation and population migration rate (0.47) (Fig 4). Taken together, the spatial changes in the health level of older adults in the YREB are the result of the combined effects of the natural environment and social environment factors.

(2) Stepwise regression analysis was used to analyze the intensity of influencing factors on the health level of older adults in the YREB, and the results revealed that annual precipitation (*r* = 0.23), average altitude (*r* = -0.18), population migration rate (*r* = 0.21), per capita GDP (*r* = 0.18), number of health institutions per 1000 population (*r* = -0.15), and household support rate (*r* = -0.21) had a significant impact on the health level of older adults (*y* = 0.23*x*_*2*_-0.18*x*_*3*_+0.21*x*_*4*_+0.18*x*_*5*_-0.15*x*_*6*_-0.21*x*_*11*_). In theory, the economic support of family members is helpful for the life of the elderly, and sufficient healthcare resources are also more conducive to timely medical treatment for the elderly. However, the results of this study show a negative correlation, which indicates the complex relationship between household support rate, healthcare resource level, and the health level of older adults, the following will discuss these relationships. Overall, the health level of older adults is closely related to natural environment, population mobility, social development level, and family characteristics.

### 4.2 Spatiotemporal differentiation of influencing factors

**Fig 5:**
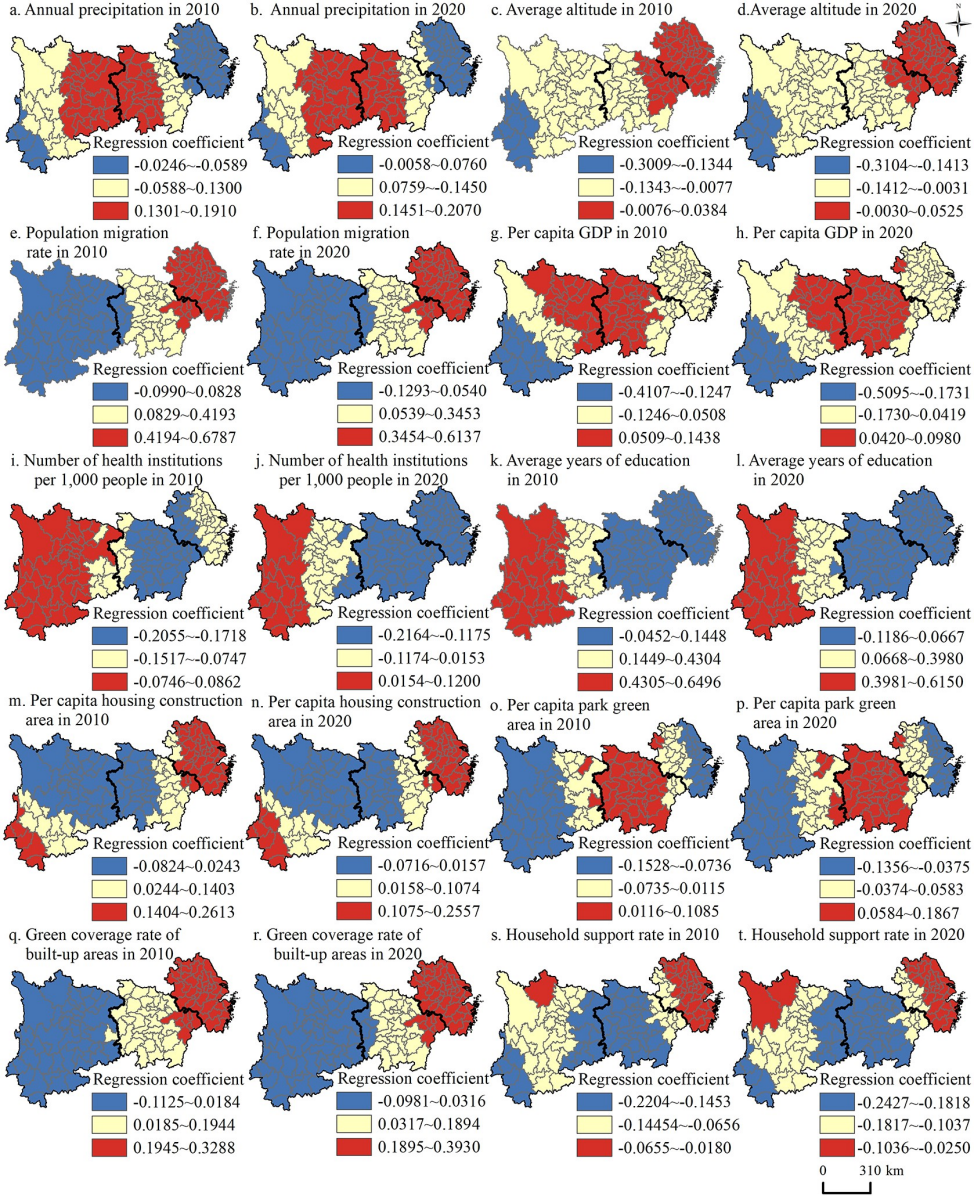
Spatiotemporal changes in factors influencing the health level of older adults in the Yangtze River Economic Belt, 2010–2020. Note: The basemap was obtained from the United States Geological Survey (https://apps.nationalmap.gov/services/), and the map boundary has not been changed. Cartographic software: ArcGIS.

From the above analysis, the average annual temperature did not pass the factor intensity screening. Therefore, only the remaining 10 influencing factors were included in the GTWR model. By drawing the time distribution map of the coefficients of these 10 factors on the health level of older adults, we aim to explore the mechanism changes of the five dimensions of natural environmental, population migration, social development level, living environment, and family characteristics. The *R*^*2*^ values of the goodness of fit and the *AICc* of the GTWR model are 0.561 and -437.1, respectively, indicating an excellent fit. The regression results are shown in Table 3 and Fig 5.

**Table 3:** GTWR results of factors affecting the health level of older adults in the Yangtze River Economic Belt.

Influencing factors		The YREB		Upstream region		Midstream region		Downstream region	
		2010	2020	2010	2020	2010	2020	2010	2020
Natural environmental	Annual precipitation	0.094	0.115	0.127	0.147	0.135	0.156	0.016	0.040
	Average altitude	-0.023	-0.022	-0.080	-0.085	-0.007	-0.008	0.024	0.033
Population migration	Population migration rate	0.269	0.214	-0.059	-0.089	0.282	0.228	0.623	0.540
Social development level	Per capita GDP	0.015	0.0003	-0.035	-0.084	0.077	0.063	0.009	0.030
	Number of health institutions per 1000 people	-0.114	-0.125	0.003	0.001	-0.192	-0.207	-0.167	-0.184
	Average years of education	0.168	0.097	0.462	0.402	0.018	-0.055	-0.011	-0.093
Living environment	Per capita housing construction area	0.080	0.060	0.021	0.021	0.025	0.011	0.202	0.151
	Per capita park green area	-0.034	0.020	-0.081	-0.027	0.055	0.115	-0.072	-0.021
	Green coverage rate in built-up areas	0.124	0.127	-0.036	-0.048	0.145	0.124	0.283	0.326
Family characteristics	Household support rate	-0.111	-0.149	-0.134	-0.164	-0.165	-0.204	-0.030	-0.077

#### 4.2.1 Natural environment. The health improvement effect of natural environmental factors increased from 2010 to 2020, and the influence of annual precipitation on shaping the spatial pattern of older adults’ health level became more obvious

As depicted in Table 3, the average value of the influencing coefficient of natural environmental factors on the health level of older adults in the YREB increased from 0.035 in 2010 to 0.047 in 2020, and the overall health improvement effect on the health level of older adults increased, indicating that the ability of older adults to adjust and adapt to the local natural conditions has largely increased over time. Compared with the average altitude, the annual precipitation exerted a more significant impact on shaping the spatial pattern of the health level of older adults. Subregionally (Fig 5), the influencing coefficient of annual precipitation from 2010 to 2020 is positive, and the effect intensity shows a spatial differentiation pattern of midstream region > upstream region > downstream region, with mean values of annual precipitation in the upstream, midstream, and downstream regions of 1,083.68 mm, 1,367.57 mm, and 1,449.4 mm, respectively, and the average values of older adults’ health levels are 0.53, 0.58, and 0.67, respectively, showing that moderate humidity is more conducive to the health level of older adults. Due to the lower annual precipitation in the upstream and midstream regions, the increase in annual precipitation in these regions has a more significant health promoting effect on the older adults than in the downstream region. The overall effect of the average altitude from 2010 to 2020 on the health level of older adults is lower. The altitude in the midstream and upstream regions has a negative impact on the health level of older adults, while the altitude in the downstream region is beneficial to the health level of older adults. The average altitude in the upstream, midstream and downstream regions are 1410.65m, 309.53m, and 108.38m, respectively. It can be seen that higher altitude is not conducive to the health status of older adults, mainly because the climate and environment in higher altitude areas have special characteristics, which require higher physical health requirements. As one ages, the body’s functions gradually decline, including the heart, lungs, and immune system. In high-altitude areas, oxygen is scarce and the oxygen content in the air is low, which can bring greater physiological burden to the elderly and easily lead to breathing difficulties, cardiovascular problems, and so on. In addition, the temperature changes in high-altitude areas are significant, with a large temperature difference between day and night. For elderly people whose body regulation function is not as good as that of young people, it can easily lead to physical discomfort and health problems. Therefore, in the Yangtze River Basin, areas with lower altitude and moderate humid climates are more conducive to the health of older adults.

#### 4.2.2 Population migration. The influencing coefficient of population migration on the health level of older adults in the YREB decreased from 0.269 in 2010 to 0.214 in 2020. Although the overall promoting effect on the health level of older adults tends to weaken, it is the primary factor influencing the spatiotemporal differentiation of the older adults’ health level in the YREB

From a subregional perspective (Fig 5), the influencing coefficient of the population migration rate in the upstream region is distinctly negative, with its absolute value increasing from 0.059 in 2010 to 0.089 in 2020, and the inhibitory effect tends to strengthen. The influencing coefficients of the midstream and downstream regions are positive, declining from 0.282 and 0.623 in 2010 to 0.228 and 0.540 in 2020, respectively, and the promoting effect tends to weaken. The effect intensity shows a pattern of high and low gradient differentiation, with the downstream region being strong, followed by the midstream region, and the upstream region being the weakest. From 2010 to 2020, the average population migration rates in the upstream, midstream, and downstream regions were -10.42%, -9.63%, and -0.89%, respectively, and the average values of older adults’ health level were 0.53, 0.58, and 0.67, respectively. The population migration rate and the health level of older adults in the upstream region are notably lower than those in the midstream and downstream regions, signifying a significant outflow of the population from the upstream region, a decrease in the size of traditional families and an increase in the number of left-behind elderly people, thereby reducing the quality of life and the health level of older adults [6]. Conversely, the influx of a large number of young and middle-aged people in downstream regions accelerates the process of regional urbanization, which helps residents better utilize basic services such as health care and education, brings convenience to the lives of elderly people, and promotes their self-assessed health [48].

#### 4.2.3 Social development level. The mean value of the influencing coefficient of the social development level on the health level of older adults in the YREB changed from 0.023 in 2010 to -0.010 in 2020, which indicates an overall shift from a positive health effect (improvement) to a negative health effect (loss)

The per capita GDP denotes the regional economic standard, and economic growth is the material basis for safeguarding population health, which affects the health level of older adults via multiple channels, such as regional health care, education, and social security [49]. The influencing coefficient of per capita GDP decreased from 0.015 to 0.0003 from 2010 to 2020, reflecting a weakening of the marginal health effect of economic growth. The effect intensity showed a spatial differentiation pattern of midstream region > upstream region > downstream region. From 2010 to 2020, the mean per capita GDP in the upstream, midstream, and downstream regions were 33,500 yuan, 43,500 yuan, and 67,300 yuan, respectively, and the average health level of older adults were 0.53, 0.58, and 0.67, respectively. The gradient difference between per capita GDP and health level is consistent, indicating that economic development could positively contribute to bolstering the health level of older adults, with this effect being most obvious in the upstream and midstream regions. Although the per capita GDP of downstream region is the highest, the promoting effect is relatively smallest, so it can be seen that the health promoting effect of economic growth does not predictably increase in a linear fashion.

The number of health institutions per 1,000 people characterizes the effect of regional medical level on the health level of older adults, and the influencing coefficient is negative, with the absolute value increasing from 0.114 to 0.125, suggesting that medical level may have a negative effect on improving the health level of older adults, and the degree of effect tends to increase. Subregionally (Fig 5), the effect intensity of the number of health institutions per 1,000 people on the health level of older adults from 2010 to 2020 shows a gradient differentiation of midstream region > downstream region > upstream region. The average number of health institutions per 1,000 people in the upstream, midstream, and downstream regions from 2010 to 2020 were 0.77, 0.63, and 0.42 per thousand people, respectively, and the average health level of older adults were 0.53, 0.58, and 0.67, respectively. It is not difficult to see that the differences between the high and low gradients are exactly the opposite, implying that a decrease in the number of health institutions per 1,000 people is counterproductive to improving the health level of the older adults in the region. Due to the large number of health institutions per 1000 people in the upstream region, increasing the allocation of health institutions per 1000 people has a health improvement effect on the health level of older adults, while the lower number of health institutions per 1,000 people in the midstream and downstream regions have significant loss effect on the health level of older adults. This suggests that the current allocation of medical resources in the YREB is unreasonable and cannot promote the health level of older adults, the midstream and downstream regions have the most significant effect.

The average years of education symbolizes the level of regional education, which can affect population health by enhancing the health literacy and awareness of the population, optimizing health behavior, and increasing investment in social health factors. The influencing coefficient decreased from 0.168 in 2010 to 0.097 in 2020, suggesting that increasing the education is beneficial to the health level of older adults, but the degree of its effect tends to weaken over time. In terms of subregions (Fig 5), the effect intensity of average years of education from 2010 to 2020 shows greater in upstream region than in midstream and downstream regions. It is worth noting that the influencing coefficient of average years of education in the downstream region is negative, with an absolute value increasing from 0.011 in 2010 to 0.093 in 2020. The mean average years of education in the upstream, midstream, and downstream regions from 2010 to 2020 are 8.09, 9.16, and 9.05 years, respectively, reflecting that the improvement in the regional education level might have adverse effects on the health level of older adults in the downstream region, because the education level in the downstream region is already superior, and the health-promoting effect derived from extending the years of education is negligible. Moreover, the influencing coefficient in the midstream region has also changed from positive to negative, indicating that the health-promoting effect of the increase in years of education has shown a significant downward trend. The highly educated elderly population has a greater demand for improving their personal health, indicating out that the health level of older adults in the region should be promoted through other strategies, such as improving the social security system. The influencing coefficient of average years of education in the upstream region is positive, exhibiting the maximum level of effect. This is attributed to the fact that the average years of education in the upstream region is less than that in the midstream and downstream regions, so the health promotion effect of the increase in years of education in the upstream region is greater than that in the midstream and downstream regions.

#### 4.2.4 Living environment. The mean value of the influencing coefficient of the living environment in the YREB climbed from 0.057 to 0.069 from 2010 to 2020, showing that its effect on improving the health level of older adults increased

The per capita housing construction area reflects the impact of living conditions on the health level of older adults. Inadequate living conditions, such as overcrowding, dampness and darkness, and poor construction quality, are often accompanied by an increased incidence of respiratory diseases, chronic diseases, and psychological disorders, thereby negatively affecting the health of the population [50]. The influencing coefficient of the per capita housing construction area in the YREB decreases from 0.080 in 2010 to 0.060 in 2020, which means that the expansion of the per capita housing construction area contributes positively to improving the health level of older adults, but this promoting effect tends to weaken. At the subregional level (Fig 5), from 2010 to 2020, the effect intensity of the per capita housing construction area on the health level of older adults in the YREB decreased in the following order: downstream region > midstream region > upstream region. The mean values of per capita housing construction area in the upstream, midstream, and downstream regions were 37.44, 42.69, and 41.86 square meters/person, respectively, and the average health level of older adults were 0.53, 0.58, and 0.67, respectively. A higher per capita housing construction area in the midstream and downstream regions corresponds to a higher health level of older adults, and the health-promoting effect of an increase in per capita housing floor area is more significant than that in the upstream region.

The per capita park green area is the main area where seniors perform daily activities, and it is also an essential indicator that reflects the living environment and quality of residence of urban residents. The influencing coefficient of the per capita park green area in the YREB from 2010 to 2020 changed from negative to positive, increasing from -0.034 to 0.020. In terms of subregions (Fig 5), the influencing coefficient of the per capita park green area in the upstream and downstream regions changed from -0.081 and -0.072 in 2010 to -0.027 and -0.021 in 2020, with its health loss effect on the health level of older adults weakening. The influencing coefficient of the midstream region increased from 0.055 to 0.115, and its health-promoting effect on the health level of older adults increased significantly. The mean per capita park green areas in the upstream, midstream, and downstream regions from 2010 to 2020 were 11.47, 12.18, and 13.87 square meters/person, respectively, which indicated that the per capita park green area in the upstream region was the smallest and had a strongest negative impact on the health level of older adults. The health level of older adults in downstream region is also negatively correlated with the per capita park green area, but the health loss effect tends to weaken. This may be due to the fast pace of urban life and high building density in downstream region, resulting in limited daily activity spaces for older adults. Therefore, their demand for surrounding park green areas is higher. Overall, parks are currently the main place for retired elderly people in China to engage in cultural and entertainment activities nearby, meeting their physiological, psychological, social and other needs. Increasing the per capita park green area to improve the health level of the elderly requires time, and this positive impact on the health level of older adults is becoming increasingly evident.

An increase in the green coverage rate in built-up areas is conducive to improving the urban ecological environment and upgrading the quality of the living environment. The influencing coefficient of the green coverage rate in the built-up areas of the YREB changed from 0.124 in 2010 to 0.127 in 2020, which promoted the health level of older adults. Subregionally (Fig 5), from 2010 to 2020, the effect intensity of the green coverage rate in built-up areas in the upstream, midstream, and downstream regions decreased in the following order: downstream region>midstream region >upstream region. The mean values of the green coverage rate in built-up areas in the upstream, midstream, and downstream regions were 36.03%, 41.55%, and 41.46%, respectively, and the average health level of older adults were 0.53, 0.58, and 0.67, respectively. Both the high and low gradient differences are basically the same, suggesting that the increase in the green coverage rate in urban built-up areas is beneficial for improving the health of older adults, especially in downstream regions.

#### 4.2.5 Family characteristics

**The household support rate reflects the impact of relying on other family members for support later in life on the health level of older adults. When the main source of livelihood is family support, it will have a significant negative effect on the mental health of older adults, particularly urban older adults and male older adults [39]. The influencing coefficient of the household support rate from 2010 to 2020 is negative, and the absolute value increases from 0.111 to 0.149, implying that household support has an inhibitory effect on the health level of older adults and tends to strengthen.** The effect intensity of the household support rate from 2010 to 2020 revealed the following trend: midstream region > upstream region > downstream region. The household support rates in the upstream, midstream, and downstream regions were 41.85%, 41.6%, and 35.83%, respectively, and the health levels of older adults were 0.53, 0.58, and 0.67, respectively. It is not difficult to determine that the gradients of the two gradients are opposite. In the midstream and upstream regions, more elderly people mainly depend on other family members for assistance, but their health levels are lower, indicating that relying on other family members for assistance in the later years of one’s life is not conducive to improving the health level of older adults. For the first time, data from the seventh National Population Census showed that, retirement pensions became the main source of livelihood for elderly people in China, especially for the urban elderly population. The economic independence of older adults has a significant positive effect on their self-rated health [51]. For the downstream region with a higher urbanization rate, although the household support rate is the lowest, the resulting adverse health effects are minimal. Therefore, it is necessary to broaden the coverage of social pension security, optimize the allocation of health care resources and social pension service resources, and improve the health security of regional older adults from the perspective of social support.

## 5. Discussion

Based on the health data of older adults in the YREB from 2010 to 2020, the temporal changes, spatial patterns and clustering characteristics, as well as the intensity and mechanism changes of influencing factors in the health level of older adults in the YREB, were analyzed through exploratory spatial data analysis, center of gravity model, geographical detector, stepwise regression analysis, and GTWR. The health level of older adults is the result of the joint effect of the natural environment and social environment factors, with social environmental factors being the main factor.

### (1) Spatiotemporal characteristics of the health level of older adults

The spatiotemporal pattern analysis of the health level of older adults in the YREB finds that the overall health level of older adults in the YREB slightly increased from 2010 to 2020. The development of health level of older adults is uneven among regions, which in the downstream region is greater than that in the midstream and upstream regions. The center of gravity of older adults’ health has always been east of the geometric center, which also confirms this conclusion. Studies on the self-rated health status of the elderly population in various provinces of China have revealed that the self-rated health status of the urban elderly population decreases from east to center to west, while the self-rated health status of the rural elderly population increases in the southern region and decreases in the northern region [6], and the overall self-rated health status of the elderly population in Jiangxi Province and the Yangtze River Delta region is excellent [5], which is basically consistent with the results of our study. This study also found that the cold spots in the health level of older adults in the YREB have shifted from upstream to midstream region, mainly due to the decline in the health level of older adults in Hubei and Hunan provinces. This conclusion is inconsistent with previous studies, as the health indicators of older adults in this study are more objective and diversified, which also provides reference for the health research of older adults in Hubei and Hunan provinces.

### (2) The influencing factors of the health level of older adults

Regarding the natural environmental factors affecting the health level of older adults, this study found that an increase in annual precipitation has a positive effect on the health level of older adults, while the average altitude is inversely proportional to the health level of older adults. Therefore, areas with low altitude and humid climates are more conducive to the health level of older adults in the YREB, which is consistent with studies in Australia [52], the United States [53], and Nepal [54]. Weather and climate changes are among the most active determinants of population health in the natural environment. Moderate precipitation can increase air humidity and have long-term beneficial effects on respiratory diseases [55]. A study in South Australia showed that the elderly population has a better self-rated health status between indoor temperatures of 18.4°C to 24.3°C and 55% relative humidity [52]. As one ages, the body’s functions gradually decline, including the heart, lungs, and immune system. The climate environment in high-altitude areas has special characteristics, such as low air pressure, scarce oxygen, and low oxygen content in the air, which can bring significant physiological burden to the elderly and easily lead to breathing difficulties, cardiovascular problems, and so on. In addition, the temperature changes in high-altitude areas are significant, with a large temperature difference between day and night. For older adults whose body regulation function is not as good as that of young people, it is more likely to lead to physical discomfort and health problems. Study in the Nepal found that a positive correlation between altitude and hypertension, as well as overweight/obesity [54]. Moreover, rapidly rising to high altitudes may adversely effects on the cardiovascular health of lowland people, especially those with previous illnesses. Sudden cardiac death (SCD) is the most common cause of nontraumatic death in males who engage in leisure activities such as downhill and hiking in high-altitude areas [56].

Population migration has a promoting effect on the health level of older adults in the YREB. In other words, the large-scale influx of middle-aged and young people into the area can promote regional economic growth, increase fiscal revenue, and enhance the convenience of medical treatment, education, entertainment and other activities for residents, thereby improving the physical and mental health of older adults. This is particularly evident in the midstream and downstream regions of the YREB. At the same time, the floating population, mainly the young and middle-aged labor force, has a low utilization rate of public medical resources [6], which will be more conducive to improving the health level of the areas where the population flows into. However, due to the migration of middle-aged and young people, the number of left-behind elderly people in the outflow areas has increased, and traditional intergenerational relationships between families have been disrupted, causing adverse effects on the psychological health of older adults. The main manifestation is that population migration in the upstream region of the YREB is inversely proportional to the health level of older adults, and this effect has increased over time.

Social environment factors can affect the health level of older adults through income, medical security, education, living circumstances, population migration, and social support. In this study, the health level of older adults in the YREB is directly proportional to the per capita GDP; that is, areas with better economic levels usually have more healthy elderly adults. The average number of years of education positively affects the health level of older adults in the YREB. These findings suggest that the health level of older adults in regions with higher economic levels, per capita income, and education levels are generally better, which is similar to findings of studies in western Hunan [27], Shanghai [57], Indonesia [58], and South African [59]. Compared with previous studies or known hypotheses on the health status of older adults, there are also inconsistencies in this study. An increase in the average years of education in the midstream and downstream regions of the YREB had an adverse effect on the health level of older adults. The reason for this may be the greatly developed economy in the midstream and downstream regions, where the education level of older adults is already higher, and improving the education level in the region cannot directly improve the health level of older adults. On the other hand, this may also be due to the greater demand of highly educated elderly people for improving their own health. Therefore, additional methods, such as improving the social security system, should be used to continue improving the health level of older adults in downstream regions. In addition, the number of health institutions per 1,000 people has negative effect on the health level of older adults in the YREB, and this effect has an increasing trend, which is inconsistent with expectations. Effective and accessible health care is a key factor in achieving healthy aging in the region [60]. In the United States, having health insurance and having access to regular sources of health care can improve health status and reduce mortality in general among older adults [61]. In China, insufficient health care services among older adults are significantly associated with increased all-cause mortality, especially in rural areas [62]. From this, it can be inferred that the reason for the negative correlation may be that the overall supply of medical and health resources in the YREB is insufficient and cannot meet the growing health needs of older adults, especially in midstream and downstream regions where the number of health institutions per 1,000 people is the lower, and where the adverse impact on the health level of older adults is the stronger. Therefore, it is necessary to strengthen health care reform, increase the supply of medical resources, and expand medical insurance coverage to promote the health level of older adults.

Improvement of the living environment in the YREB is conducive to enhancing the health level of older adults. Specifically, the per capita housing construction area is directly proportional to the health level of older adults; that is, improving of housing conditions is conducive to improving the self-evaluated health status of the elderly population, which is similar to the findings of studies in Japan [63], the United States [64], and Sweden [65]. On the one hand, elderly people have a strong emotional attachment to the houses in which they have lived for a long time. On the other hand, due to their weak physical functions and mobility, their daily activities are also mainly conducted at home. Therefore, poor living conditions, a lack of independent living space and basic living facilities can have long-term adverse effects on the physical and mental health of older adults [27]. An increase in the per capita park green area and green coverage rate in built-up areas in the YREB are beneficial for improving the health level of older adults, which is consistent with the research findings of Shanghai [18] and Bangladesh [66]. Numerous studies have suggested that green space is considered the lungs of a city and that the presence of green space has immense health benefits, especially for elderly people [67]. On the one hand, urban green space can reduce the heat island effect and prevent dust and noise to improve the liveability of the living environment. On the other hand, urban green space also provides public entertainment space for residents. Regular physical exercise in green space considerably reduces the risk of obesity, cardiovascular disease, respiratory disease, high blood pressure, diabetes and other chronic diseases and increases the social interactions of older adults, thereby to promoting their physical and mental health [68].

The higher the household support rate of older adults in the YREB is, the lower their health level. Due to the current incomplete social security service system in China, the family elderly care model is the main choice for older adults in current and future periods. Adult children bear the vital responsibility of providing economic support, emotional comfort, and daily care for their parents. Therefore, intergenerational family support can alleviate the pressure of aging and social pressure to some extent and has a positive impact on the health and social stability of older adults [20]. Different forms of intergenerational support have different impacts on the health status of older adults. The emotional comfort and daily care of adult children significantly improve effect on the physical health of elderly people, and the health-promoting effect of daily care on the older adults is significantly greater than that of emotional support, especially in rural elderly populations [69]. However, this paper revealed that an increase in the household support rate is not conducive to improving the health level of older adults in the YREB, especially in the upstream region. Here, the household support rate refers to the economic support of children for the older adults. That is, excessive financial support from children increases the depression in older adults and has a negative impact on their life satisfaction, which is similar to the findings of multiple studies in Europe [70], China [69], and other countries. The main reason for this phenomenon is that when older adults believe that they are in good physical condition and have sufficient economic income, they often do not need help from others (unless they are unable to take care of themselves). Economically independent elderly people, especially urban elderly people, usually have better physical and mental health [20]. Older adults with poor health conditions generally hope to receive comprehensive support from their children. In the YREB, the household support rate of older adults in the upstream region is greater, and long-term dependence on children for economic support has adverse effects on the psychological health of older adults. The household support rate of older adults in the downstream region is the lower, and its adverse effect is smaller. This also indirectly indicates that the elderly population in downstream regions is economically independent and that there are fewer elderly people who require financial support from their children, with the least adverse impact on their health. Therefore, measures such as strengthening family elderly care support, health and medical support, and social care support and improving the social security system should be taken to enhance the economic security of older adults, thereby improving their health. These conclusions have important reference significance for analyzing the factors influencing the health level of old adults among other regions.

## 6. Conclusions

In this paper, based on the data of life expectancy of the elderly, self-rated health rate of the elderly, and disability rate of the elderly, the spatiotemporal characteristics and influencing factors of the health level of older adults in the YREB during 2010–2020 were analyzed. The findings of this paper will supplement and improve the research on the health level of old adults, which has profound significance. On the one hand, it can provide reference for the research on the health of old adults in other countries and regions around the world, and on the other hand, it can also provide a basis for the research on the health of old adults in cities and counties under the YREB. At the same time, it also helps to comprehensively improve the physiological and psychological health levels of old adults in the YREB, narrow the health gap between regions and cities, realize healthy aging in the YREB, and improve and enhance the human health. The results are as follows:

According to the analysis of spatiotemporal patterns, the health level of older adults in the YREB has slightly improved from 2010 to 2020, with the most significant improvement in the upstream region and the most significant decline in the midstream region. There are obvious spatial differentiation in the health level of older adults in the upstream, midstream, and downstream regions of the YREB, with the health level of older adults in the downstream region being greater than that in the midstream and upstream regions. The hot spots of older adults’ health level in the YREB were gathered in Jiangsu, Zhejiang, Anhui, Jiangxi provinces of midstream and downstream regions, while the cold spots were mainly distributed in Yunnan, Hubei, and Hunan provinces, and showed a tendency to migrate from upstream to midstream region.

The health level of older adults in the YREB is influenced by a combination of natural environment and social environment factors. Average years of education, population migration rate, average altitude, per capita GDP, household support rate, number of health institutions per 1000 population, green coverage rate in built-up areas, per capita park green area, annual precipitation, per capita housing construction area, which have a higher impact on the health level of old adults in the YREB. In terms of interaction detection, the interaction between socio-economic factors and natural meteorological factors is significantly stronger. Moreover, annual precipitation, per capita GDP, and population migration have a promoting effect on the health level of older adults, while average altitude, number of health institutions per 1000 population, and household support rate have an inhibitory effect on the health level of older adults.

Regarding the mechanism changes of factors influencing the health level of older adults in the YREB, the health-promoting effect of natural environmental factors is enhanced, and the influence of annual precipitation on shaping the spatial pattern of older adults’ health level became more obvious. Although the promoting effect of population migration on the health level of older adults tends to weaken, it remains the primary factor affecting the spatiotemporal differentiation of older adults’ health level in the YREB. The social development level has changed from a positive effect (improvement) to a negative effect (loss) on the health level of older adults. The health-promoting effect of living environment factors is enhanced. The health-inhibitory effect of household support rate is increased and showed a gradient decreasing pattern from downstream to midstream to upstream.

## 7. Suggestions

Based on the above analysis of the spatiotemporal patterns of the health level of older adults in the YREB and the factors influencing it, in order to improve the health level of older adults, reduce the disparities between cities and regions, and realize healthy aging and active aging in the YREB, this study suggests that: firstly, comprehensively consider various factors that affect the health level of older adults, such as economy, health resources, education, social security, living environment, natural conditions, personal and family characteristics, and make concerted efforts to build a wall to ensure the health level of older adults in the YREB. In addition, it is necessary to focus on aspects that affect the health level such as health resources, living environment, and family support, optimize the allocation level of medical resources in the YREB, improve the efficiency of medical resource utilization, and increase the supply of urban medical security resources, as well as continuously expand the coverage of urban and rural basic old-age pension and basic medical insurance, and push forward the reform of the social security system. Improve the quality of living environment, by strengthening the structure and spatial planning of urban construction land, coordinating the development and flow of urban population, increasing investment in the construction of urban green areas and providing government support. These will meet the growing demand for recreational activities among urban residents, enhance urban liveability, in order to realize older adults with medical care, care for elderly people, living in a house, living in a place of joy, and to comprehensively improve the health level of older adults. Secondly, the spatial differences in the impact of various factors on the health level of older adults should be taken into account, so as to formulate differentiated strategies for the development of elderly health. Specifically, the upstream region should vigorously improve their education level, continue to increase the urban green coverage, improve the social security systems, and strive to overcome the limitations of natural conditions caused by low temperature and drought on the basis of developing regional economy. In the midstream and downstream regions of the YREB, while continuing to improve the regional economic level, the supply of medical and healthcare resources should be increased, and urban parks and green areas should be constructed and developed, so as to achieve an overall improvement in the health level of the elderly people.

## Supporting information

S1 File (PDF)
