# Supplementary material for: Geographical characteristics and influencing factors of the health level of older adults in the Yangtze River Economic Belt, China, from 2010 to 2020
Source: PLoS One. 2024 Sep 13;19(9):e0308003. doi: 10.1371/journal.pone.0308003 (PMC11398639; doi:10.1371/journal.pone.0308003)
Supplement: S1 File — (PDF) [file pone.0308003.s001.pdf]

Geographical characteristics and influencing factors of the health level of older adults in the Yangtze River Economic Belt, China, from 2010 to 2020

Mengmeng Yang , Shengsheng Gong

Supplementary Material

1. Health level of older adults in the Yangtze River Economic Belt

In this work, the Yangtze River Economic Belt (YREB) includes 11 provinces and 131 prefecture level units, as shown in Figure 1. According to the watershed division, the YREB can be divided into upstream, midstream, and downstream regions. Among them, the upstream region includes 47 prefecture-level units under the jurisdiction of the four provinces and municipalities of Yunnan, Guizhou, Sichuan and Chongqing. The middle-stream region includes 42 prefecture-level units under the jurisdiction of the three provinces of Hubei, Hunan and Jiangxi. The downstream region includes 42 prefecture-level units under the jurisdiction of the four provinces and municipalities of Jiangsu, Zhejiang, Anhui and Shanghai. The health levels of older adults in the YREB, upstream region, midstream region, and downstream region from 2010 to 2020 are shown in Table 1. The life expectancy of the elderly, self-rated health rate of the elderly, and disability rate of the elderly in various prefecture level cities are shown in Table 2. Based on these data, the spatiotemporal evolution characteristics of the health level of older adults in the YREB were obtained through exploratory spatiotemporal analysis, as shown in Figure 2-3.

2. Analysis of influential factors of older adults' health level in the Yangtze River Economic Belt

The influencing factors of the health level of older adults in the YREB from 2010 to 2020 are shown in Table 3. Based on data on the health level of older adults and influencing factors, the effect intensity of the influencing factors was analyzed using geographical detector and stepwise regression analyses. The analysis results are shown in Figure 4. The Spatio-temporal geographically weighted regression (GTWR) is used to analyze the mechanism changes of influencing factors on the health level of older adults in the YREB. See Table 4 and Figure 5 for analysis results.

Table 1 Temporal changes of older adults' health level in the Yangtze River Economic Belt

Time	The Yangtze River Economic Belt	The upstream region	The midstream region	The downstream region
2010	0.588	0.507	0.593	0.675
2020	0.592	0.556	0.564	0.661

Table 2 The life expectancy of the elderly, self-rated health rate of the elderly, and disability rate of the elderly of 131 prefecture level units in the Yangtze River Economic Belt, from 2010 to 2020

Name	2010			2020		
	Disability rate of the elderly(%)	Self-rated health rate of the elderly (%)	Life expectancy of the elderly (Year)	Disability rate of the elderly(%)	Self-rated health rate of the elderly (%)	Life expectancy of the elderly (Year)
chengdu	1.51	91.89	23.52	2.03	92.23	26.37
zigong	2.76	81.50	22.23	2.22	84.94	25.69
panzhihua	3.45	82.89	23.03	2.67	86.89	24.86
luzhou	2.64	82.06	21.15	2.16	86.13	25.25
deyang	2.27	85.62	21.97	2.09	87.78	23.94
mianyang	3.15	81.55	21.32	2.32	86.36	25.25
guangyuan	5.41	77.83	20.18	2.83	81.98	24.11
suining	3.50	75.68	23.62	2.52	84.61	26.93
neijiang	2.65	79.23	22.22	2.08	85.15	25.04
leshan	2.64	82.71	22.91	2.34	86.19	24.77
nanchong	5.40	71.80	23.12	2.86	80.83	25.91
meishan	2.81	81.90	22.67	2.36	85.85	24.44
yibin	2.96	79.81	20.31	2.78	85.12	23.47
guangan	3.37	77.54	20.56	2.13	84.46	25.24
dazhou	3.72	78.16	20.77	1.96	84.41	25.69
yaan	3.61	81.49	23.00	2.51	87.97	24.77
bazhong	6.66	74.48	23.39	2.97	80.01	26.08
ziyang	2.93	79.52	21.73	2.62	82.64	25.57
aba	3.96	81.14	22.38	2.43	87.15	25.87
ganzi	5.65	74.52	21.55	3.45	83.28	26.15
liangshan	3.84	83.29	20.43	2.44	87.70	26.18
chongqing	3.12	81.42	22.72	1.96	90.11	24.15
kunming	2.88	86.54	20.39	2.05	89.28	24.30
qujing	3.23	83.64	20.06	1.91	86.35	22.46
yuxi	3.36	83.93	21.38	2.65	84.44	23.18
baoshan	4.08	82.43	20.59	2.33	88.40	23.15
zhaotong	6.00	75.63	20.03	2.50	84.44	21.59
lijiang	5.76	77.03	20.43	2.59	85.10	23.50
puer	4.70	77.81	20.38	2.94	84.36	22.94
lincang	4.23	82.39	19.93	2.38	88.69	21.62
chuxiong	4.19	81.64	19.44	2.41	85.04	21.43
honghe	3.81	82.17	19.55	2.02	88.33	22.11
wenshan	3.24	83.29	18.99	1.79	88.69	22.58
xishuangbanna	3.42	86.03	21.33	2.52	88.15	22.91
dali	3.71	86.19	20.20	2.12	89.66	22.30
dehong	3.59	83.26	20.48	3.24	86.10	23.18
nujiang	5.21	82.61	20.12	4.60	78.47	22.86
diqing	3.56	80.98	20.62	3.77	81.81	22.41
guiyang	2.30	89.33	23.20	1.66	92.83	23.97
liupanshui	3.96	78.91	21.32	1.84	92.10	22.36
zunyi	3.73	79.63	20.95	1.90	89.74	23.01
anshun	2.54	83.75	21.35	1.75	92.11	22.06
bijie	4.59	76.23	21.12	2.10	89.76	24.30
tongren	4.02	76.71	21.72	2.06	88.34	23.18
qianxinan	3.18	81.41	21.50	1.78	92.19	23.26
qiandongnan	2.77	80.61	20.84	2.08	89.51	22.84

qiannan	2.46	82.95	21.23	1.73	92.24	23.63
wuhan	2.23	85.96	22.90	1.89	89.19	23.31
huangshi	2.85	81.91	22.20	2.46	86.90	22.85
shiyan	5.90	74.46	22.41	2.47	82.34	23.23
yichang	3.55	78.38	22.58	2.18	86.59	24.13
xiangyang	2.97	83.47	22.02	2.15	86.97	24.41
ezhou	1.86	83.24	20.55	1.95	88.10	22.94
jingmen	2.32	84.15	22.06	1.87	88.39	23.29
xiaogan	2.12	79.83	21.04	1.63	85.74	22.06
jingzhou	2.39	81.53	21.48	1.68	87.01	25.30
huanggang	3.35	74.05	22.10	2.20	82.17	23.68
xianning	4.03	74.47	23.08	2.47	80.41	23.46
suizhou	2.93	76.36	23.33	2.11	82.45	24.73
enshi	4.82	73.73	21.22	2.19	84.89	23.77
xiantao	1.70	85.38	21.31	0.99	90.02	21.81
qianjiang	2.09	85.21	22.91	1.26	90.80	23.47
tianmen	1.96	83.16	19.51	1.17	86.87	20.72
shennongjia	7.19	71.08	23.37	3.57	77.00	22.80
changsha	2.30	84.06	22.17	2.25	88.59	22.25
zhuzhou	2.73	82.91	21.87	2.42	87.57	23.48
xiangytan	2.63	82.01	22.09	2.46	85.22	22.44
hengyang	2.95	77.76	21.24	1.95	86.70	23.49
shaoyang	3.12	75.31	21.46	2.38	84.01	22.10
yueyang	3.29	77.81	22.04	2.03	86.49	22.94
changde	3.02	78.41	23.63	2.04	85.47	24.09
zhangjiajie	3.98	78.66	23.00	2.78	81.50	23.76
yiyang	3.74	75.23	23.00	2.32	82.22	22.55
chenzhou	2.91	80.67	23.07	1.98	87.47	21.75
yongzhou	2.49	80.88	22.39	2.02	86.10	22.11
huaihua	2.65	80.82	23.36	2.12	87.44	23.35
loudi	3.94	73.99	21.33	2.57	82.18	22.76
xiangxi	2.49	82.14	21.97	2.21	85.26	22.18
nanchang	1.73	89.82	21.82	1.74	92.65	25.49
jingdezhen	1.92	87.31	18.94	1.63	90.93	23.76
pingxiang	2.33	87.03	21.39	2.01	90.16	23.26
jiiujiang	2.94	81.45	21.32	1.71	89.77	25.33
xinyu	1.80	88.73	22.74	1.62	90.49	25.51
yingtan	1.84	90.71	21.21	1.42	91.97	23.76
ganzhou	2.08	85.05	21.13	1.57	91.17	24.13
jian	2.18	85.23	22.16	1.69	89.66	23.65
yichun	2.07	85.43	21.19	1.85	90.78	23.88
fuzhou	1.79	86.52	19.38	1.32	91.00	23.62
shangrao	2.40	83.49	20.50	1.58	90.33	24.24
hangzhou	2.33	89.21	23.47	2.11	92.64	26.49
ningbo	2.25	89.00	23.32	1.82	92.30	26.12
wenzhou	2.95	85.73	22.19	2.24	90.68	24.59
jiaxing	1.58	92.06	22.50	1.61	94.33	25.74
huzhou	1.75	90.64	22.36	1.55	93.88	25.65
shaoxing	2.11	89.21	22.03	1.58	92.62	25.61
jinhua	2.66	86.35	22.34	2.49	88.66	25.75
quzhou	2.21	86.67	22.42	2.51	87.89	25.16
zhoushan	2.91	72.85	22.37	1.62	93.13	25.54
taizhou	2.68	86.88	22.39	2.28	90.64	25.27
lishui	2.58	85.42	22.25	2.64	86.22	25.29
hefei	2.73	85.65	23.61	2.28	89.21	26.59
chaohu	2.40	83.35	23.61	2.34	86.24	26.59
wuhu	2.26	86.30	21.54	2.20	87.30	23.81
bengbu	3.24	78.63	22.38	2.49	86.15	25.08
huainan	3.44	81.12	23.36	2.52	86.38	25.62
maanshan	2.41	85.10	22.92	2.51	87.38	24.99
huaibei	3.89	76.26	24.48	3.56	81.89	26.38
tongling	2.74	85.85	21.66	2.54	85.82	23.75
anqing	3.78	74.87	22.12	2.32	88.06	23.65
huangshan	3.08	79.66	22.36	2.19	87.02	24.37

chuzhou	3.69	77.87	22.43	2.37	87.01	24.86
fuyang	3.75	77.08	21.01	2.96	82.65	24.95
suzhou	4.04	77.01	22.91	2.69	84.33	24.95
liuan	3.58	76.83	21.12	2.45	85.95	24.36
bozhou	4.11	75.56	21.60	3.27	81.39	25.41
chizhou	3.30	80.27	21.60	2.49	85.89	23.82
xuancheng	2.58	82.74	21.51	2.65	84.81	23.72
nanjing	2.49	88.38	23.15	2.13	92.01	26.75
wuxi	2.08	91.29	23.07	2.07	93.06	25.30
xuzhou	2.70	82.94	21.48	1.97	88.26	24.48
changzhou	2.09	90.15	22.85	1.99	92.36	25.65
suzhou	1.64	92.29	23.54	1.33	95.32	26.82
nantong	2.96	86.80	22.86	2.60	88.89	25.35
lianyungang	2.70	83.20	21.09	1.90	88.49	24.27
huaian	2.57	83.69	21.19	2.07	88.43	23.62
yancheng	2.69	85.21	21.55	1.94	87.83	24.45
yangzhou	1.75	91.31	21.33	1.47	92.69	24.24
zhenjiang	1.74	91.29	21.94	1.82	91.37	24.40
taizhou	2.24	88.11	22.41	1.87	90.42	25.37
suqian	2.89	79.91	21.08	2.65	84.01	23.61
shanghai	3.71	87.27	24.38	3.71	87.27	26.51

Table 3 Influencing factors of the Yangtze River Economic Belt from 2010 to 2020

Name	Annual average temperature (x1/℃)	Annual precipitation (x2/mm)	Average altitude (x3/m)	Population migration rate (x4/%)	Per capita GDP (x5/Yuan/person)	Number of health institutions per 1,000 people (x6/Number)	Average years of education (x7/Year)	Per capita housing construction area (x8/ Square meters per person)	Per capita park green area (x9/ Square meters per person)	Green coverage rate in built-up areas (x10/%)	Household support rate (x11/%)	The health level of older adults	Time
aba	9.20	831.80	3626.76	-0.33	14662	1.73	7.59	34.77	10.24	5.53	55.46	0.55	2010
anqing	17.00	2044.10	195.06	-16.49	18647	0.15	8.03	38.52	9.70	38.48	52.08	0.45	2010
anshun	15.60	1278.40	1204.72	-20.50	10014	0.74	7.28	26.04	1.23	14.00	40.30	0.62	2010
bazhong	16.70	1297.00	800.88	-19.96	8717	0.93	8.20	31.71	8.84	35.03	37.37	0.35	2010
bengbu	15.60	801.30	21.23	-15.55	20223	0.13	8.50	30.66	7.03	37.00	40.13	0.56	2010
baoshan	16.40	1041.80	1816.67	-1.71	10469	0.15	7.69	34.03	7.86	31.00	57.48	0.46	2010
bijie	14.00	883.10	1686.43	-27.53	9113	0.41	6.71	21.44	0.49	8.50	42.55	0.36	2010
bozhou	15.50	695.10	31.20	-25.81	10615	0.05	7.43	33.73	10.92	39.53	39.64	0.41	2010
changde	18.10	1590.00	190.48	-8.95	26551	0.24	11.16	39.44	14.10	43.35	40.01	0.64	2010
changzhou	16.50	1085.00	14.68	21.49	67327	0.24	9.57	39.58	12.35	42.15	37.90	0.83	2010
chaohu	16.40	1316.80	30.73	-18.98	48312	0.14	7.63	33.20	7.74	37.44	43.29	0.75	2010
chenzhou	19.30	1181.00	506.36	-9.36	24015	0.21	8.97	28.03	8.03	36.97	48.00	0.65	2010
chengdu	16.00	936.80	826.05	18.66	41253	0.51	9.93	36.58	13.21	39.43	34.09	0.94	2010
chizhou	17.00	1850.40	186.99	-13.85	21476	0.17	8.02	40.61	18.08	39.14	50.80	0.53	2010
chuzhou	16.10	1205.30	48.58	-15.13	17693	0.09	7.90	32.63	12.67	36.24	49.22	0.52	2010
chuxiong	17.00	612.50	1927.76	1.71	14960	0.21	8.06	29.85	13.73	32.40	48.58	0.37	2010
dazhou	17.40	1046.20	680.72	-24.86	14623	0.74	8.11	31.78	14.43	35.76	39.98	0.43	2010
dali	16.50	679.50	2241.89	-1.92	13498	0.20	7.98	28.72	7.01	34.77	56.36	0.52	2010

dehong	20.10	1206.40	1413.81	5.05	11681	0.28	7.54	26.88	8.21	25.40	50.52	0.50	2010
deyang	16.30	841.20	807.22	-7.02	25335	0.76	8.44	35.60	9.65	38.27	32.26	0.70	2010
diqing	9.10	464.80	3462.12	10.49	20051	0.24	7.19	36.99	1.24	1.10	45.73	0.47	2010
ezhou	16.93	1386.91	31.57	-3.24	37928	0.18	9.09	41.24	14.13	37.00	39.69	0.60	2010
enshi	15.88	1044.05	1074.24	-20.83	10327	0.14	8.30	46.81	10.06	33.68	40.55	0.32	2010
fuzhou	18.70	2409.70	232.31	-5.11	16083	0.12	8.52	31.53	16.62	48.01	49.61	0.59	2010
fuyang	15.50	655.30	32.13	-33.46	9528	0.07	7.61	34.01	7.51	33.02	38.41	0.42	2010
ganzi	8.10	955.90	4181.20	3.17	11659	2.10	5.78	26.45	3.44	4.14	46.81	0.30	2010
ganzhou	19.90	1326.60	370.55	-9.13	13397	0.19	8.49	30.72	12.18	45.09	58.04	0.65	2010
guangan	17.50	972.30	410.33	-45.60	15588	0.96	7.76	38.52	15.31	41.67	44.93	0.42	2010
guangyuan	16.40	1095.60	916.75	-24.56	12313	1.36	7.98	39.20	8.88	36.56	42.33	0.28	2010
guiyang	14.40	977.40	1188.08	13.59	26209	0.68	9.61	28.66	10.06	37.20	30.66	0.83	2010
hangzhou	17.40	1728.10	272.31	20.96	69828	0.32	10.08	41.53	15.12	39.95	32.67	0.84	2010
hefei	16.40	1316.80	41.75	11.98	48312	0.14	10.08	30.01	13.21	38.82	32.60	0.77	2010
hengyang	19.10	1137.00	160.86	-10.54	20419	0.09	8.50	33.83	9.21	38.95	48.47	0.49	2010
honghe	19.40	952.80	1487.64	0.90	14546	0.53	7.31	24.82	9.37	28.60	49.77	0.41	2010
huzhou	16.50	1312.40	109.90	10.19	45323	0.46	8.47	45.66	15.28	49.78	45.17	0.83	2010
huaihua	18.10	1431.00	436.96	-7.40	14371	0.25	8.83	30.81	8.12	30.38	48.11	0.69	2010
huaian	15.00	931.80	14.48	-12.97	28861	0.46	8.78	35.90	10.97	39.60	50.00	0.60	2010
huaibei	15.80	642.50	32.70	-4.20	22309	0.17	8.75	31.58	13.26	43.16	42.75	0.61	2010
huainan	16.80	1000.80	25.72	-5.52	26287	0.24	8.91	29.81	11.46	39.83	35.60	0.64	2010
huanggang	16.41	1309.28	175.31	-20.78	13322	0.14	8.24	42.34	11.12	32.97	43.47	0.46	2010
huangshan	17.00	2187.20	380.71	-8.70	22791	0.39	8.40	29.39	14.52	48.82	38.97	0.58	2010
huangshi	17.13	1477.22	108.89	-10.32	28481	0.14	9.10	36.66	11.95	39.88	38.61	0.62	2010
jian	19.10	2208.70	256.34	-3.61	14969	0.14	8.64	32.92	13.36	42.22	50.63	0.71	2010
jiaxing	16.80	1354.00	6.53	23.74	52143	0.31	8.49	48.03	12.92	41.09	32.14	0.87	2010
jinhua	18.10	2137.60	297.16	13.36	39897	0.32	8.72	42.00	12.15	39.80	45.88	0.71	2010
jingmen	16.82	957.85	120.76	-4.20	25462	0.21	9.36	35.92	10.26	39.92	42.74	0.68	2010
jingzhou	17.00	1150.49	43.38	-16.67	14707	0.16	8.81	34.94	9.51	39.79	40.72	0.60	2010

jingdezhen	18.00	2571.50	144.11	1.12	29155	0.24	8.88	34.80	15.65	53.57	36.48	0.57	2010
jiujiang	17.90	1743.40	209.28	-5.47	21686	0.17	9.07	32.82	18.11	56.39	48.89	0.56	2010
kunming	16.80	603.90	2098.52	15.83	33549	0.47	9.52	31.74	8.36	41.36	32.89	0.58	2010
leshan	17.50	1380.80	1085.58	-9.49	22490	0.91	8.42	38.66	7.10	37.91	38.41	0.69	2010
lijiang	15.10	821.10	2581.85	2.84	11680	0.56	7.94	28.21	13.86	56.08	56.06	0.27	2010
lishui	18.50	2089.70	627.67	-18.01	31296	0.16	8.11	38.28	10.48	41.33	49.86	0.70	2010
lianyungang	14.00	867.70	15.85	-13.42	26987	0.60	8.90	34.05	12.02	38.73	44.52	0.58	2010
liangshan	17.80	1084.00	2643.88	-5.85	17560	1.09	6.41	27.09	4.00	38.07	44.37	0.48	2010
lincang	19.20	1055.20	1629.21	2.16	8988	0.15	6.93	22.05	13.73	35.22	53.66	0.41	2010
liuan	16.00	1507.30	185.26	-26.85	12074	0.09	7.95	32.10	12.02	40.79	44.08	0.43	2010
liupanshui	14.60	1198.40	1715.15	-10.42	17462	0.51	7.40	23.87	2.44	24.70	40.44	0.46	2010
loudi	17.30	1334.00	354.71	-14.24	17569	0.14	9.16	33.41	9.07	39.79	53.41	0.38	2010
luzhou	17.70	1021.70	705.32	-19.57	16698	0.97	7.89	35.78	8.31	39.00	43.26	0.57	2010
maanshan	16.70	1232.40	17.64	5.24	60712	0.22	9.12	30.49	13.96	42.73	32.61	0.74	2010
meishan	17.30	1028.20	696.39	-18.27	18586	0.67	8.15	42.83	11.83	31.75	46.76	0.65	2010
mianyang	16.70	884.40	1229.58	-16.51	20053	0.86	8.37	40.25	10.39	37.89	33.31	0.55	2010
nanchang	18.50	2211.10	38.99	1.11	43961	0.15	10.32	31.01	9.01	42.76	34.76	0.79	2010
nanchong	17.60	1068.00	411.61	88.01	13212	1.28	8.08	34.48	8.65	38.12	42.21	0.37	2010
nanjing	16.20	1298.40	25.00	20.48	65273	0.28	10.99	34.10	13.69	44.38	27.50	0.80	2010
nantong	15.70	1356.80	4.01	-5.15	48083	0.47	8.83	50.04	10.50	40.60	34.08	0.73	2010
neijiang	17.30	1183.20	403.96	-15.55	18022	0.80	8.23	30.46	6.43	34.64	44.12	0.59	2010
ningbo	17.40	1476.30	138.05	24.43	69368	0.31	9.04	34.51	10.49	38.04	32.89	0.83	2010
nujiang	16.70	720.60	2780.45	0.63	10266	0.11	6.54	19.68	5.19	10.20	46.56	0.37	2010
panzhihua	21.50	725.70	1851.93	7.92	43959	0.84	8.71	28.31	8.22	40.55	37.50	0.65	2010
pingxiang	18.30	1968.20	316.58	-2.94	28106	0.14	9.26	35.77	12.07	46.72	56.56	0.68	2010
puer	19.70	1355.30	1429.01	1.42	9584	0.16	7.17	21.15	2.22	37.07	46.13	0.34	2010
qianjiang	17.34	1115.37	29.84	-8.85	30719	0.31	8.82	33.40	10.10	40.02	33.87	0.76	2010
qiandongnan	16.30	1143.70	765.94	-28.03	8839	0.95	7.41	28.97	10.30	33.50	46.82	0.52	2010
qiannan	16.50	1236.40	992.64	-23.12	10861	0.65	7.46	30.25	8.64	39.00	46.35	0.60	2010

qianxinan	17.10	1270.00	1156.16	-18.53	10839	0.12	7.11	28.56	3.45	35.40	48.12	0.55	2010
qujing	15.10	739.70	2010.32	-7.16	17228	0.10	7.47	26.66	9.29	38.93	48.43	0.50	2010
quzhou	17.50	2412.50	336.53	-17.39	35500	0.33	8.09	56.05	13.07	42.17	45.88	0.75	2010
shanghai	17.40	1317.30	4.56	38.38	76074	0.14	10.73	27.25	6.97	38.15	4.92	0.79	2010
shangrao	17.80	2767.20	213.49	-13.72	13729	0.12	8.21	36.06	15.38	48.35	54.64	0.57	2010
shaoyang	18.00	1243.00	560.76	-12.10	10468	0.10	8.83	29.56	8.42	32.97	47.41	0.46	2010
shaoxing	17.70	1494.40	185.44	10.59	57580	0.30	8.81	44.90	15.38	40.35	45.30	0.77	2010
shennongjia	16.29	841.78	1670.24	-8.08	16347	0.25	8.22	37.35	2.65	3.75	42.59	0.27	2010
shiyan	15.12	826.09	732.24	-5.72	21267	0.25	8.67	30.80	10.00	45.32	39.29	0.34	2010
suzhou	17.00	931.90	4.52	39.03	93043	0.26	9.85	39.61	16.86	42.70	21.41	0.94	2010
suqian	14.70	1041.30	14.08	-17.61	22525	0.61	8.29	33.95	12.14	40.57	54.41	0.52	2010
suzhou	15.80	630.30	32.83	-19.36	12195	0.07	8.11	32.45	10.52	38.02	41.33	0.52	2010
suizhou	16.00	880.75	188.23	-18.92	18381	0.07	8.75	34.16	10.27	31.98	42.47	0.60	2010
suining	16.90	1146.90	362.19	-16.43	14498	1.14	8.17	34.64	7.55	37.66	42.14	0.57	2010
taizhou	17.80	2408.90	249.03	2.39	41172	0.23	8.08	40.11	10.61	44.27	49.77	0.72	2010
taizhou	16.10	1144.90	2.96	-8.49	44118	0.41	8.59	47.89	9.34	40.82	36.82	0.77	2010
tianmen	17.34	1092.85	31.22	-17.67	15468	0.13	8.54	37.35	5.84	35.38	37.29	0.53	2010
tongling	17.00	1532.70	53.73	-2.47	64496	0.22	9.11	35.72	10.93	40.17	32.27	0.66	2010
tongren	16.60	1245.10	751.61	-35.44	9304	0.94	7.56	31.85	3.65	34.00	48.41	0.44	2010
wenzhou	18.30	2435.40	356.92	13.91	32586	0.50	8.40	31.22	6.04	21.89	52.25	0.67	2010
wenshan	18.10	778.70	1361.69	-5.11	9456	0.05	7.24	27.88	2.69	13.64	45.82	0.43	2010
wuxi	16.90	931.10	17.15	26.68	92167	0.31	9.81	39.86	14.41	42.62	34.35	0.87	2010
wuhu	16.80	1343.80	38.41	-1.97	49013	0.20	9.17	30.83	9.45	38.20	32.51	0.69	2010
wuhan	16.60	1264.21	39.97	14.32	65920	0.28	11.12	33.74	8.89	37.17	18.87	0.76	2010
xishuangbanna	8.10	421.20	1099.00	16.61	14503	0.06	7.10	26.78	13.27	39.50	54.63	0.60	2010
xiantao	17.36	1228.38	26.68	-31.19	18732	0.30	8.58	39.98	11.75	41.40	40.94	0.69	2010
xianning	17.39	1449.16	188.73	-20.37	21002	0.13	8.43	37.53	9.34	37.60	46.30	0.49	2010
xiangtan	17.90	1550.00	111.07	-4.95	32305	0.36	8.94	41.53	8.72	40.38	41.80	0.63	2010
xiangxi	17.40	1735.00	548.72	-11.62	11991	0.26	8.27	28.14	6.00	26.40	48.45	0.63	2010

xiangyang	15.90	810.29	348.17	-7.70	26026	0.55	9.07	35.75	10.75	36.38	35.86	0.63	2010
xiaogan	16.58	1035.65	78.73	-8.98	15096	0.11	8.67	33.93	10.06	40.21	40.78	0.56	2010
xinyu	18.90	2169.60	125.27	-2.08	55492	0.19	9.38	34.80	15.80	49.17	39.69	0.82	2010
xuzhou	15.20	612.00	33.06	-13.79	34084	0.49	8.92	34.11	14.74	41.26	42.80	0.60	2010
xuancheng	16.20	1519.10	207.08	-9.68	20779	0.15	7.86	35.76	14.13	35.12	46.44	0.61	2010
yaan	16.40	2092.30	2099.22	-2.84	18881	0.85	8.46	36.10	15.50	39.81	44.09	0.62	2010
yancheng	14.70	968.20	1.34	-12.96	31640	0.39	8.72	34.02	11.68	39.20	38.26	0.64	2010
yangzhou	16.00	1119.80	6.58	-3.84	49786	0.45	9.03	41.88	19.14	43.60	43.06	0.78	2010
yibin	18.30	1019.60	573.53	-20.90	19499	0.93	7.94	35.84	15.96	38.98	41.33	0.47	2010
yichang	16.69	1002.22	661.13	1.50	38824	0.33	9.40	41.63	10.88	40.88	37.14	0.55	2010
yichun	18.00	2137.00	219.98	-3.88	10675	0.15	8.82	33.25	14.52	42.46	48.54	0.66	2010
yiyang	18.00	1537.00	202.81	-10.48	16710	0.16	9.27	34.36	7.58	40.17	51.49	0.51	2010
yingtan	18.60	2643.40	167.02	-4.60	30769	0.33	8.60	39.78	12.69	47.34	48.72	0.76	2010
yongzhou	18.70	1291.00	423.66	-17.39	14853	0.18	8.94	30.73	5.64	31.35	49.54	0.64	2010
yuxi	18.00	672.80	1667.13	5.93	32089	0.32	8.04	35.70	10.14	34.35	48.65	0.57	2010
yueyang	16.10	1648.00	129.86	-3.15	28849	0.22	9.58	34.13	8.51	41.37	49.95	0.52	2010
zhangjiajie	17.70	1582.00	545.66	-11.37	16238	0.22	8.94	40.03	7.68	37.72	39.51	0.55	2010
changsha	18.50	1554.00	170.71	7.51	66464	0.36	10.48	42.47	9.98	36.19	45.66	0.69	2010
zhaotong	16.10	740.10	1697.17	-11.82	7193	0.09	7.00	20.47	29.90	35.34	48.72	0.21	2010
zhenjiang	16.20	1298.50	23.00	12.66	64284	0.28	9.58	40.00	15.95	42.14	41.62	0.82	2010
chongqing	18.70	1044.70	733.52	-14.92	28084	0.09	8.75	36.99	13.24	40.57	36.50	0.63	2010
zhoushan	16.70	1367.80	58.85	13.51	58378	0.36	8.62	34.68	15.08	40.24	48.38	0.49	2010
zhuzhou	18.50	1625.00	309.37	-1.07	33604	0.35	9.35	44.71	12.70	42.41	48.55	0.62	2010
ziyang	17.10	931.50	409.62	-37.18	16644	1.37	7.83	36.87	5.70	35.00	39.13	0.55	2010
zigong	17.80	978.00	370.37	-21.49	23613	0.79	8.18	32.78	8.07	38.10	38.84	0.62	2010
zunyi	15.60	1095.50	984.60	-25.00	14650	0.78	7.82	31.68	4.94	34.60	49.82	0.46	2010
aba	9.50	1037.90	3626.76	-8.99	49668	2.10	7.75	48.74	12.72	20.78	47.90	0.66	2020
anqing	17.30	2136.70	195.06	-26.76	58684	0.45	8.93	49.14	15.15	39.49	53.44	0.57	2020
anshun	15.80	1583.30	1204.72	-24.29	39160	0.81	7.94	45.50	24.60	41.30	47.17	0.61	2020

bazhong	16.60	936.00	800.88	-35.06	27951	1.23	8.73	45.43	15.24	41.68	40.19	0.49	2020
bengbu	16.30	1101.30	21.23	-17.06	63209	0.73	8.78	44.32	14.63	43.06	34.12	0.60	2020
baoshan	16.80	1 251.7	1816.67	-8.64	43200	0.58	8.24	47.30	11.02	38.92	54.12	0.55	2020
bijie	13.90	1126.90	1686.43	-35.94	29295	0.80	7.43	38.15	15.91	40.89	50.02	0.65	2020
bozhou	16.50	908.50	31.20	-32.60	36156	0.22	8.05	55.36	18.06	39.89	38.52	0.45	2020
changde	17.00	1554.90	190.48	-14.39	70496	1.75	9.23	49.81	14.19	44.51	36.41	0.57	2020
changzhou	17.40	1085.80	14.68	26.77	147939	0.30	10.23	45.55	14.33	43.26	21.19	0.78	2020
chaohu	16.20	1523.00	30.73	-13.79	108427	0.37	9.04	42.96	13.56	45.00	38.01	0.69	2020
chenzhou	18.10	1397.40	506.36	-14.10	53581	1.59	9.09	43.82	13.59	46.56	41.29	0.49	2020
chengdu	16.80	1229.60	826.05	28.40	85679	0.57	10.51	41.67	14.12	43.75	17.34	0.82	2020
chizhou	17.20	2065.20	186.99	-21.12	64843	1.72	8.56	51.85	18.97	46.13	54.01	0.52	2020
chuzhou	16.20	1323.20	48.58	-14.11	76087	0.27	9.45	38.96	19.31	42.30	38.18	0.61	2020
chuxiong	17.60	701.50	1927.76	3.64	56433	0.74	8.54	44.14	14.42	38.06	42.63	0.39	2020
dazhou	18.10	1089.40	680.72	-22.26	38068	0.84	8.56	41.03	14.20	40.00	37.28	0.64	2020
dali	16.80	790.60	2241.89	-9.20	44346	0.62	8.53	46.69	12.39	39.16	48.14	0.54	2020
dehong	20.30	1 470.9	1413.81	0.15	43817	0.43	8.23	41.28	12.53	44.00	47.12	0.42	2020
deyang	17.20	1132.10	807.22	-10.98	69443	0.71	8.77	45.17	14.15	42.27	22.43	0.60	2020
diqing	8.60	651.60	3462.12	4.39	68711	0.75	7.65	51.89	10.60	35.21	45.22	0.25	2020
ezhou	17.28	912.16	31.57	-3.46	93074	0.44	9.57	49.90	16.18	43.23	32.09	0.57	2020
enshi	17.48	862.31	1074.24	-16.38	32303	0.86	8.98	56.39	17.89	43.32	34.59	0.53	2020
fuzhou	18.90	2185.20	232.31	-19.61	43305	0.73	8.68	51.79	17.90	50.27	41.27	0.71	2020
fuyang	16.50	1047.80	32.13	-31.38	34399	0.21	8.18	53.80	17.73	39.54	38.28	0.48	2020
ganzi	7.90	968.20	4181.20	1.99	36993	2.54	6.74	39.31	10.75	25.42	40.97	0.51	2020
ganzhou	20.90	1415.40	370.55	-9.59	40754	0.97	9.09	53.67	13.64	49.85	46.80	0.72	2020
guangan	17.60	1074.00	410.33	-41.23	40073	1.03	8.25	45.12	16.88	42.72	37.15	0.61	2020
guangyuan	16.40	1107.00	916.75	-29.44	43337	1.46	8.50	48.13	16.19	40.15	32.54	0.44	2020
guiyang	14.80	1427.60	1188.08	28.55	72246	0.55	10.28	39.88	17.71	40.69	23.73	0.73	2020
hangzhou	18.20	1632.90	272.31	31.98	136617	0.47	10.76	44.52	12.27	43.36	14.42	0.82	2020
hefei	16.20	1523.00	41.75	17.78	108427	0.37	10.39	36.91	12.67	41.99	33.30	0.75	2020

hengyang	19.50	1159.80	160.86	-20.03	52550	1.29	9.06	48.03	13.75	42.62	45.45	0.57	2020
honghe	19.60	1 140.5	1487.64	2.01	53925	0.53	8.12	40.10	7.92	57.32	41.68	0.52	2020
huzhou	17.70	1798.40	109.90	20.50	95579	0.44	9.21	52.46	17.85	46.33	22.96	0.85	2020
huaihua	17.40	1805.80	436.96	-14.41	36365	2.20	8.80	44.26	8.74	39.48	45.30	0.56	2020
huaian	15.50	859.20	14.48	-22.38	87507	0.50	9.18	48.75	14.34	42.60	40.09	0.60	2020
huaibei	15.90	992.70	32.70	-11.02	56661	1.62	9.03	46.49	18.48	45.25	34.68	0.49	2020
huainan	16.30	1267.10	25.72	-28.98	43557	0.71	8.88	40.52	13.79	39.81	31.33	0.63	2020
huanggang	16.83	888.46	175.31	-26.85	36898	0.72	8.62	53.94	13.77	42.49	44.36	0.47	2020
huangshan	17.80	2456.90	380.71	-11.95	63940	2.04	8.93	54.13	17.13	48.03	35.07	0.60	2020
huangshi	17.49	951.56	108.89	-10.72	66449	0.58	9.19	48.58	16.94	40.42	34.63	0.49	2020
jian	20.00	2219.90	256.34	-20.81	48307	0.97	8.71	60.49	17.24	46.42	38.62	0.66	2020
jiaxing	17.90	1638.00	6.53	32.10	102541	0.32	9.34	50.14	15.75	39.34	20.16	0.86	2020
jinhua	19.30	1609.40	297.16	30.06	67329	0.61	9.38	45.96	12.29	41.54	30.29	0.68	2020
jingmen	16.88	725.91	120.76	-13.74	70162	0.25	9.98	48.89	13.58	41.12	31.68	0.60	2020
jingzhou	17.30	852.99	43.38	-22.60	45296	0.58	9.03	48.99	12.56	37.01	30.75	0.70	2020
jingdezhen	19.00	2116.80	144.11	-5.62	59134	0.70	9.06	56.98	17.49	53.96	25.34	0.69	2020
jiujiang	17.50	2047.60	209.28	-14.13	70341	0.58	9.31	55.18	15.41	48.83	37.81	0.75	2020
kunming	17.00	850.10	2098.52	31.68	80586	0.65	10.22	41.95	12.05	42.15	28.16	0.65	2020
leshan	18.30	1555.30	1085.58	-10.76	63259	1.02	8.72	47.45	14.86	42.19	24.27	0.59	2020
lijiang	15.60	1 009.2	2581.85	2.38	40841	0.65	8.57	44.11	17.58	40.06	47.07	0.48	2020
lishui	19.40	1239.30	627.67	-7.95	61811	0.66	8.88	48.79	12.07	42.82	32.64	0.59	2020
lianyungang	15.00	952.70	15.85	-16.17	71303	0.60	9.51	52.53	15.02	42.29	30.94	0.65	2020
liangshan	18.10	1182.50	2643.88	-9.69	35720	1.11	7.16	33.01	6.92	35.00	35.46	0.69	2020
lincang	19.30	1 183.1	1629.21	-6.64	36213	0.60	7.65	39.82	9.12	37.42	46.82	0.47	2020
liuan	16.70	2036.90	185.26	-34.65	37899	0.15	8.71	46.11	16.34	43.80	45.78	0.56	2020
liupanshui	13.90	1462.80	1715.15	-16.50	44224	0.57	8.04	40.84	12.01	40.77	47.59	0.62	2020
loudi	17.70	1844.60	354.71	-18.80	43913	2.09	9.23	50.77	9.59	41.07	49.46	0.39	2020
luzhou	18.00	1396.80	705.32	-19.48	50758	1.11	8.40	44.85	14.00	42.18	32.84	0.63	2020
maanshan	17.00	1543.30	17.64	-6.06	101011	0.77	9.04	39.94	15.35	46.28	33.20	0.61	2020

meishan	18.20	1292.60	696.39	-15.54	48132	0.72	8.57	52.54	14.40	43.30	36.91	0.57	2020
mianyang	17.70	1122.10	1229.58	-9.03	61936	1.00	8.94	45.62	14.07	41.52	25.46	0.62	2020
nanchang	19.10	2167.10	38.99	14.01	92697	0.44	10.52	41.06	12.27	41.30	25.58	0.81	2020
nanchong	17.70	1180.50	411.61	-29.06	42482	1.47	8.41	44.60	14.10	44.53	35.34	0.51	2020
nanjing	17.10	961.90	25.00	22.47	159322	0.37	11.36	39.65	16.09	44.69	11.63	0.82	2020
nantong	16.90	1301.70	4.01	2.17	129900	0.44	9.43	56.29	18.62	43.29	29.66	0.65	2020
neijiang	18.30	1025.80	403.96	-29.94	46228	1.14	8.51	42.21	16.62	38.17	36.50	0.61	2020
ningbo	18.40	1527.20	138.05	34.86	132614	0.50	9.70	39.17	13.95	42.23	14.20	0.83	2020
nujiang	16.30	1 225.6	2780.45	0.05	38141	0.61	7.62	32.38	12.75	23.70	46.04	0.14	2020
panzhihua	21.90	702.40	1851.93	10.74	85806	0.83	9.22	39.88	14.16	42.02	17.55	0.58	2020
pingxiang	19.10	1861.00	316.58	-10.50	53302	0.78	9.50	61.59	14.15	47.87	25.68	0.62	2020
puer	19.90	1 378.8	1429.01	-5.39	39172	0.26	7.92	32.43	12.07	40.89	43.44	0.41	2020
qianjiang	17.39	830.60	29.84	-12.34	82611	0.81	9.49	48.82	11.15	36.23	23.49	0.71	2020
qiandongnan	16.70	1546.80	765.94	-37.57	31719	1.04	7.92	43.97	10.20	34.00	57.04	0.57	2020
qiannan	16.70	1552.70	992.64	-22.82	45654	0.63	8.20	49.50	17.00	37.74	50.12	0.70	2020
qianxinan	17.00	1457.00	1156.16	-27.79	44881	0.77	8.04	45.75	13.02	39.04	53.63	0.67	2020
qujing	15.10	922.60	2010.32	-15.60	51244	0.47	8.21	41.91	13.52	38.93	43.66	0.51	2020
quzhou	18.80	2132.40	336.53	-12.76	72192	0.79	8.83	69.89	16.66	43.55	34.49	0.63	2020
shanghai	17.80	1660.80	4.56	40.69	155800	0.24	11.50	32.28	9.05	37.32	2.09	0.58	2020
shangrao	19.00	2088.40	213.49	-22.19	40391	1.01	8.85	64.71	17.86	48.91	40.61	0.71	2020
shaoyang	17.50	1460.80	560.76	-25.91	34063	1.76	9.03	52.42	14.44	43.04	47.67	0.41	2020
shaoxing	18.70	1684.30	185.44	15.40	113746	0.54	9.55	52.33	14.84	44.12	24.39	0.83	2020
shennongjia	16.30	710.98	1670.24	-17.87	46200	1.34	8.59	48.42	4.00	37.72	31.86	0.21	2020
shiyan	15.37	697.31	732.24	-7.72	59660	0.87	9.17	39.27	15.00	42.24	32.38	0.43	2020
suzhou	17.90	1278.60	4.52	41.62	158466	0.30	10.30	41.59	12.37	43.10	9.01	0.96	2020
suqian	15.90	671.60	14.08	-18.70	65503	0.49	9.00	42.75	16.74	44.99	45.12	0.46	2020
suzhou	15.50	919.70	32.83	-23.51	38368	0.27	9.08	49.32	14.84	39.67	34.18	0.54	2020
suizhou	16.31	644.01	188.23	-22.91	53554	0.64	9.12	44.71	11.45	42.63	41.73	0.54	2020
suining	17.70	1166.60	362.19	-28.37	49495	1.42	8.40	46.73	15.13	41.99	36.64	0.66	2020

taizhou	19.90	1421.20	249.03	8.41	79889	0.55	8.77	46.97	13.02	43.89	32.69	0.71	2020
taizhou	16.40	825.80	2.96	-10.07	117542	0.47	9.32	55.46	15.91	42.57	32.87	0.75	2020
tianmen	17.22	759.44	31.22	-37.14	53200	0.90	8.56	55.94	13.89	39.99	32.23	0.50	2020
tongling	17.60	1930.00	53.73	-30.23	75748	1.24	8.82	43.44	18.14	45.57	42.57	0.51	2020
tongren	16.50	1489.80	751.61	-35.15	40269	1.03	8.29	48.51	14.59	40.36	49.19	0.57	2020
wenzhou	19.80	1500.80	356.92	13.03	71766	0.61	8.88	40.32	13.75	37.85	36.94	0.67	2020
wenshan	18.30	1 082.0	1361.69	1.69	33798	0.55	7.90	48.32	8.64	38.48	51.83	0.57	2020
wuxi	17.70	1229.90	17.15	31.81	165851	0.40	10.39	43.75	14.95	43.43	14.39	0.77	2020
wuhu	17.90	1565.00	38.41	-6.79	102964	0.24	9.24	40.46	12.99	41.01	38.49	0.58	2020
wuhan	17.21	801.26	39.97	25.69	126651	0.52	11.54	39.68	14.50	42.07	12.07	0.61	2020
xishuangbanna	21.90	1 064.9	1099.00	23.30	46619	0.49	7.96	40.14	13.69	42.80	36.09	0.51	2020
xiantao	16.54	853.07	26.68	-34.57	73100	0.90	8.89	44.30	11.12	38.78	32.79	0.63	2020
xianning	17.72	973.83	188.73	-13.90	58319	0.51	9.08	50.49	15.11	39.76	41.07	0.41	2020
xiangtan	18.10	1612.30	111.07	-5.86	85911	1.39	10.95	47.11	12.23	41.91	35.96	0.44	2020
xiangxi	16.90	2106.00	548.72	6.72	29059	2.60	8.52	41.94	16.49	70.24	47.24	0.45	2020
xiangyang	16.43	633.44	348.17	-11.96	87490	0.70	9.27	48.91	15.41	44.27	25.30	0.61	2020
xiaogan	16.82	713.95	78.73	-22.89	51372	0.11	8.80	45.94	10.54	41.25	36.54	0.51	2020
xinyu	18.60	1850.60	125.27	-4.17	83505	1.02	9.41	42.22	19.89	50.70	30.03	0.78	2020
xuzhou	16.00	753.40	33.06	-14.27	80673	0.50	9.19	48.58	16.58	43.10	36.48	0.65	2020
xuancheng	17.30	1875.40	207.08	-11.52	64301	0.45	8.61	47.42	17.24	42.02	45.74	0.48	2020
yaan	17.10	1870.00	2099.22	-6.99	52366	1.08	8.80	48.63	19.33	41.30	29.49	0.61	2020
yancheng	16.00	961.30	1.34	-21.37	88731	0.49	8.93	45.68	15.19	43.60	30.67	0.64	2020
yangzhou	16.60	991.40	6.58	0.30	132784	0.43	9.48	48.69	19.95	44.67	39.85	0.76	2020
yibin	17.70	1746.00	573.53	-20.26	61182	1.09	8.63	44.88	14.53	40.53	34.49	0.46	2020
yichang	16.70	753.30	661.13	2.28	106005	0.66	9.96	48.78	12.23	41.13	23.75	0.58	2020
yichun	18.70	1565.20	219.98	-20.56	55452	0.88	9.05	57.09	15.56	48.55	34.16	0.67	2020
yiyang	18.10	1711.00	202.81	-22.86	47784	1.93	8.99	46.48	11.48	40.06	44.92	0.40	2020
yingtan	19.30	2052.70	167.02	-12.17	85263	0.74	9.34	54.28	15.73	44.03	34.86	0.73	2020
yongzhou	18.90	1403.00	423.66	-21.51	39857	2.28	8.86	48.80	11.80	38.10	48.81	0.48	2020

yuxi	18.30	720.50	1667.13	1.78	91290	0.63	8.72	57.65	14.62	41.72	45.15	0.45	2020
yueyang	18.00	1813.60	129.86	-12.87	78867	1.36	9.64	45.93	12.65	43.32	40.02	0.53	2020
zhangjiajie	16.90	1973.00	545.66	-11.18	36708	1.69	8.94	49.64	8.96	36.94	42.75	0.41	2020
changsha	17.50	1503.00	170.71	26.64	123297	0.59	11.02	43.15	11.65	41.46	33.85	0.51	2020
zhaotong	15.90	906.70	1697.17	-23.58	25255	0.41	7.76	37.57	12.26	36.03	53.15	0.38	2020
zhenjiang	16.70	926.30	23.00	16.15	131580	0.33	10.02	48.59	18.05	43.38	31.05	0.72	2020
chongqing	19.20	1181.40	733.52	-6.45	78173	0.65	9.47	41.96	16.50	43.05	30.41	0.67	2020
zhoushan	18.00	1412.50	58.85	17.00	130130	0.62	9.50	43.97	16.46	44.15	17.64	0.83	2020
zhuzhou	18.80	1803.20	309.37	-3.08	79599	1.10	9.76	54.47	13.98	42.60	38.79	0.54	2020
ziyang	18.10	913.00	409.62	-48.05	34806	1.46	8.08	48.05	15.55	38.46	36.51	0.55	2020
zigong	18.90	1115.30	370.37	-28.51	58059	0.87	8.62	42.73	14.60	44.00	29.06	0.63	2020
zunyi	15.90	1338.70	984.60	-23.90	56334	0.68	8.46	43.55	22.76	41.48	50.84	0.60	2020

Table 4 GTWR results of factors affecting the health level of older adults in the Yangtze River Economic Belt

Influencing factors		The YREB		Upstream region		Midstream region		Downstream region	
		2010	2020	2010	2020	2010	2020	2010	2020
Natural environmental	Annual precipitation	0.094	0.115	0.127	0.147	0.135	0.156	0.016	0.040
	Average altitude	-0.023	-0.022	-0.080	-0.085	-0.007	-0.008	0.024	0.033
Population migration	Population migration rate	0.269	0.214	-0.059	-0.089	0.282	0.228	0.623	0.540
Social development level	Per capita GDP	0.015	0.0003	-0.035	-0.084	0.077	0.063	0.009	0.030
	Number of health institutions per 1000 people	-0.114	-0.125	0.003	0.001	-0.192	-0.207	-0.167	-0.184
	Average years of education	0.168	0.097	0.462	0.402	0.018	-0.055	-0.011	-0.093
Living environment	Per capita housing construction area	0.080	0.060	0.021	0.021	0.025	0.011	0.202	0.151
	Per capita park green area	-0.034	0.020	-0.081	-0.027	0.055	0.115	-0.072	-0.021
	Green coverage rate in built-up areas	0.124	0.127	-0.036	-0.048	0.145	0.124	0.283	0.326
Family characteristics	Household support rate	-0.111	-0.149	-0.134	-0.164	-0.165	-0.204	-0.030	-0.077

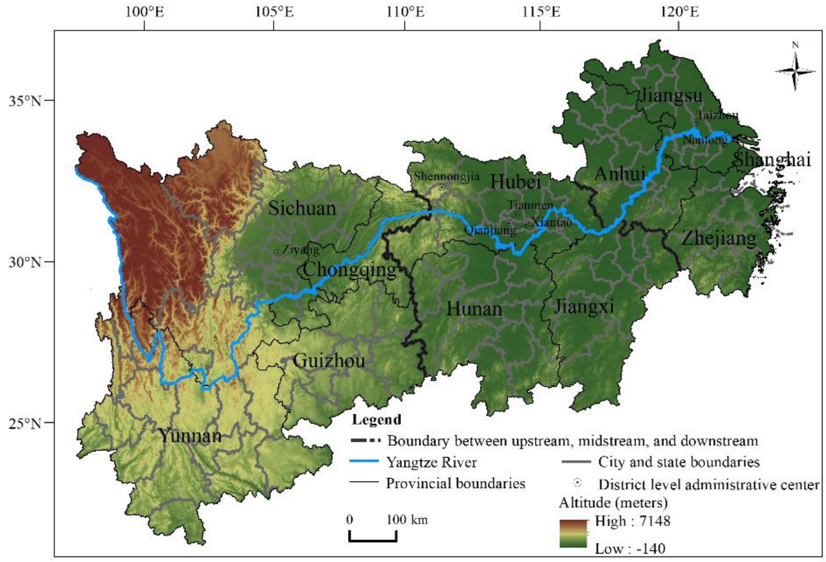

Fig 1 Overview of the Yangtze River Economic Belt

Note: The basemap was obtained from the United States Geological Survey (https://apps.nationalmap.gov/services/), and the map boundary has not been changed. Cartographic software: ArcGIS.

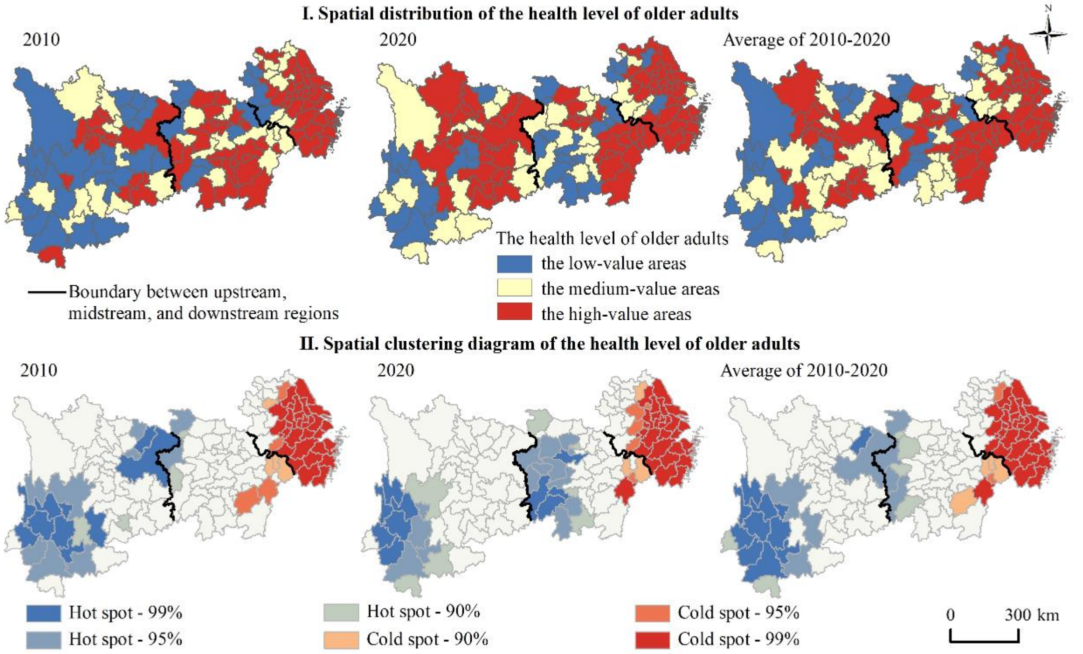

Fig 2 Spatiotemporal pattern evolution of the health level of older adults in the Yangtze River Economic Belt

Note: The basemap was obtained from the United States Geological Survey (https://apps.nationalmap.gov/services/), and the map boundary has not been changed. Cartographic software: ArcGIS.

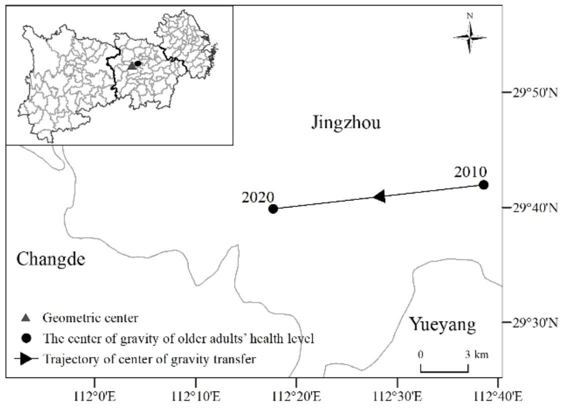

Fig 3 Migration map of the center of gravity of older adults' health level in the Yangtze River Economic Belt

Note: The basemap was obtained from the United States Geological Survey (https://apps.nationalmap.gov/services/), and the map boundary has not been changed. Cartographic software: ArcGIS.

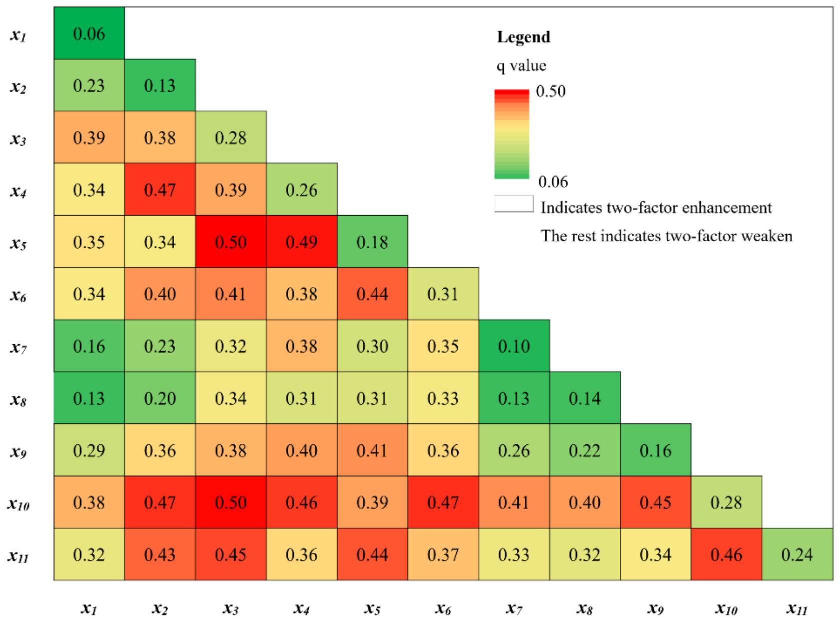

Fig 4 Interaction hotspot maps of influencing factors on older adults' health level in the Yangtze River Economic Belt

Note: x_1: average annual temperature, x_2: annual precipitation, x_3: average altitude, x_4: population migration rate, x_5: per capita GDP, x_6: number of health institutions per 1,000 people, x_7: average years of education, x_8: per capita housing construction area, x_9: per capita park green area, x_{10}: green coverage rate in built-up areas, x_{11}: household support rate.

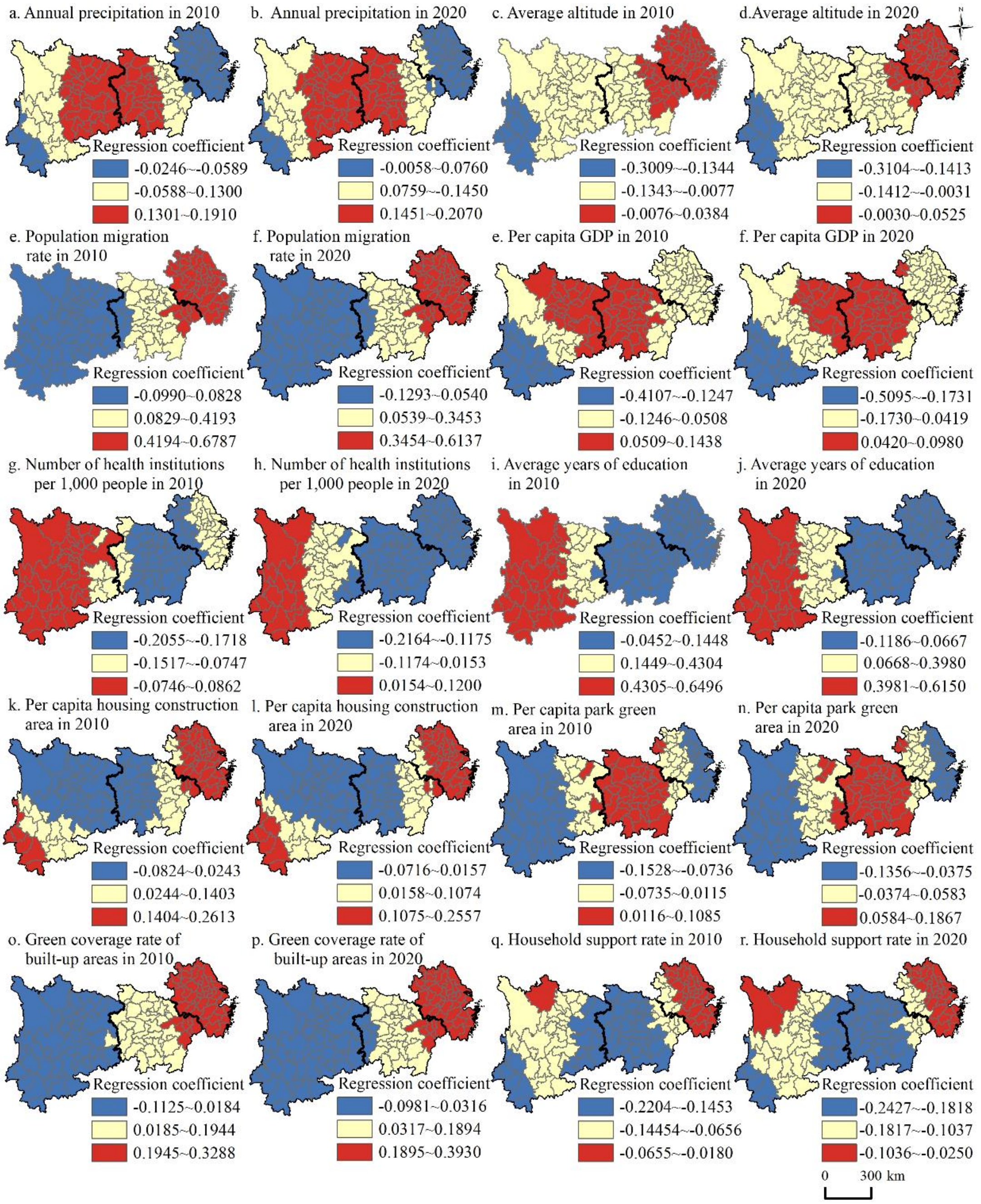

Fig 5 Spatiotemporal changes in factors influencing the health level of older adults in the Yangtze River Economic Belt, 2010-2020

Note: The basemap was obtained from the United States Geological Survey (https://apps.nationalmap.gov/services/), and the map boundary has not been changed. Cartographic software: ArcGIS.
